# Comparative genomics study of polyhydroxyalkanoates (PHA) and ectoine relevant genes from *Halomonas *sp. TD01 revealed extensive horizontal gene transfer events and co-evolutionary relationships

**DOI:** 10.1186/1475-2859-10-88

**Published:** 2011-11-01

**Authors:** Lei Cai, Dan Tan, Gulsimay Aibaidula, Xin-Ran Dong, Jin-Chun Chen, Wei-Dong Tian, Guo-Qiang Chen

**Affiliations:** 1 Dept Biological Sciences and Biotechnology, MOE Key Lab. Bioinformatics (& System Biology), Tsinghua University-Peking University Joint Center for Life Sciences, School of Life Sciences, Tsinghua University, Beijing 100084, China; 2 School of Biological Sciences and Biotechnology, Xinjiang University, Urumchi, 830046, China; 3 Institute of Biostatistics, School of Life Sciences, Fudan University, Shanghai, China

**Keywords:** *Halomonas *spp., PHB, polyhydroxyalkanoates, osmolytes, genome, PhaC

## Abstract

**Background:**

Halophilic bacteria have shown their significance in industrial production of polyhydroxyalkanoates (PHA) and are gaining more attention for genetic engineering modification. Yet, little information on the genomics and PHA related genes from halophilic bacteria have been disclosed so far.

**Results:**

The draft genome of moderately halophilic bacterium, *Halomonas *sp. TD01, a strain of great potential for industrial production of short-chain-length polyhydroxyalkanoates (PHA), was analyzed through computational methods to reveal the osmoregulation mechanism and the evolutionary relationship of the enzymes relevant to PHA and ectoine syntheses. Genes involved in the metabolism of PHA and osmolytes were annotated and studied *in silico*. Although PHA synthase, depolymerase, regulator/repressor and phasin were all involved in PHA metabolic pathways, they demonstrated different horizontal gene transfer (HGT) events between the genomes of different strains. In contrast, co-occurrence of ectoine genes in the same genome was more frequently observed, and ectoine genes were more likely under coincidental horizontal gene transfer than PHA related genes. In addition, the adjacent organization of the homologues of PHA synthase *phaC1 *and PHA granule binding protein *phaP *was conserved in the strain TD01, which was also observed in some halophiles and non-halophiles exclusively from *γ-proteobacteria*. In contrast to haloarchaea, the proteome of *Halomonas *sp. TD01 did not show obvious inclination towards acidity relative to non-halophilic *Escherichia coli *MG1655, which signified that *Halomonas *sp. TD01 preferred the accumulation of organic osmolytes to ions in order to balance the intracellular osmotic pressure with the environment.

**Conclusions:**

The accessibility of genome information would facilitate research on the genetic engineering of halophilic bacteria including *Halomonas *sp. TD01.

## Background

Polyhydroxyalkanoates (PHA) were firstly discovered in prokaryotes as carbon and energy storage materials [1]. They are one of the most promising members of biodegradable polymers, which are considered as environmentally friendly substitutes of petrochemical-derived plastics [2]. Poly-3-hydroxybutyrate (PHB) was the earliest commercially available product. However, poly(3-hydroxybutyrate-co-hydroxyvalerate) (PHBV) possessed more favourable thermomechanical properties for wider applications as medical materials (sutures and bone-nails/pins), films products (mulch films, shopping bags and compost bags), disposable items (pens and tableware) and packaging materials (especially for food packaging) than PHB [3]. As the market for green plastics has been growing rapidly, demand for a more productive and low cost PHA production process is evident [1]. Although some microorganisms, such as *Ralstonia eutropha *and genetically engineered *Escherichia coli*, were thoroughly investigated as PHA industrial producers with high productivity, the research and development of strains and methods with further reduced cost were still necessary for PHA commercialization [3-6].

Halophiles are referred to some organisms, which are able to grow optimally in 5% (w/v) and survive in no less than 10% salt medium [7,8]. Halophiles have shown advantages for lowering fermentation costs for PHB and PHBV production, thus gaining attention from many researchers. As early as 1972, PHA granules were detected in a haloarchaea *Haloarcula marismortui*, and then numerous halophiles were identified as producers to accumulate PHB or PHBV [7]. Halophiles were widely spread in the three domains of life: archaea, bacteria, and eukarya [7]. According to the optimal growth concentration of salt tolerated by cells, they could be roughly divided into two groups, moderate and extreme halophiles [9]. Recently, the application of some halophiles for PHA production and copolymer characterization were evaluated [4,7,10,11]. The application of halophiles as PHA producers significantly reduced the costs of fermentation and recovery processes: high salt concentrations were able to minimize the possibility of contamination by non-halophilic microorganisms, and thus the cost and energy consumption for sterilization can be decreased; haloarchaeal cells were able to be lyzed conveniently through osmotic shock treatment with salt-deficient water, and thus the cost for polymers recovery also can be decreased [12]. Moreover, the residual salt in the broth post-fermentation, could be concentrated and recycled, which circumvents environmental problems from the exhaust of waste products and further lowered the production cost [7]. When hydrolyzed whey was used as carbon source, the cost for the production of P(3HB-co3HV) by *Haloferax mediterranei *was about 30% lower than that for the production of PHA by recombinant *E. coli *[7]. Our recent study showed a new isolated halophilic strain termed *Halomonas *sp. TD01, has great potential for PHA production, accumulating over 80 wt% PHB under a continuous fermentation process without sterilization [13].

PHA synthases, the crucial enzymes for PHA biosynthesis, have received more attention for elucidating their characteristics in these microorganisms. PHA synthases were classified into four groups according to their substrate specificities and subunits organization [1]. Although class I and II PHA synthases comprise enzymes consisting exclusively of one PhaC subunit, they prefer to synthesize short chain length (3-5 carbon atoms) and medium chain length (6-14 carbon atoms) PHA, respectively. Class III and IV PHA synthases comprise enzymes consisting of two different types of subunits, PhaC and PhaE (III) or PhaR (IV, such as that in *Bacillus megaterium*) subunits; both of them prefer to utilize CoA thioesters to produce short chain length PHA [1]. The study on PHA synthases isolated from PHA accumulating halophiles began with the characterization of PhaC from *Halopiger aswanensis*, which showed some interesting properties including high thermostability, narrow substrate specificity and tolerance against high salt concentration [14]. A novel subclass of PhaCs was proposed through systematic and detailed studies on the PHA synthases from haloarchaea, which were in high similarity with the class III enzymes from bacteria [15]. However, the *H. mediterranei *PhaC subunit was larger, and the PhaE subunit was smaller than its bacterial counterparts [16].

Osmoregulatory mechanisms of halophiles against the high salt concentration condition were typically divided into two strategies: extreme halophiles, i.e. halophilic archaea, were able to absorb KCl to balance the osmotic pressure discrepancy across the cytoplasmic membrane; most moderate halophiles were able to accumulate osmolytes (compatible solutes), which mainly consisted of organic compounds with low molecular weight, including amino acids, amino acid derivatives, sugars, and other polyols [17]. Organic compounds did not notably disturb the essential metabolism for cell survival, and protected proteins by mitigating detrimental effects of freezing, drying and high temperatures [8]. Ectoine is one of the most widespread osmolytes, which are also well known as commercial protectants for enzymes, DNA and whole cells [18].

In this study, the draft genome of the moderately halophilic bacterium *Halomonas *sp. TD01 was obtained and analyzed. Several genes relevant to PHA and osmolytes biosynthesis were elucidated and phylogenetically analyzed. Moreover, the predicted proteome was analyzed and compared with that of other species. The results provided invaluable clues, not only to the understanding of the evolution and genes transfer, but also to the strategic guidance of the genetic engineering of halophilic *Halomonas *sp. TD01 for co-production of PHA and ectoine.

## Methods

### Strain and genome DNA preparation

*Halomonas *sp. TD01 was isolated from a salt lake in Xinjiang, China, and grown optimally in glucose mineral medium under 5% (w/v) NaCl and pH 9.0 [13]. And it was deposited in China General Microbiological Culture Collection Center (CGMCC No.4353). To construct the insert libraries for sequencing, high-quality total cellular DNA was prepared with the help of the E.Z.N.A. bacterial DNA kit (Omega Bio-Tek Inc. USA).

### Sequencing strategy

Two random genomic DNA libraries with insert sizes of 500 and 2,530 base pairs (bp) were constructed, respectively. The sequencing of these libraries was carried out following the Solexa sequencing protocols (Illumina, Inc. USA) in the Beijing Genomics Institute (BGI, Beijing, China). Eliminating the low-quality results and adapter contamination, raw data was assembled into contigs and scaffolds with SOAPdenovo software (v1.04, BGI, Beijing, China).

### Genome annotation

Glimmer 3.0 software [19] was adopted to predict genes *de novo*. For annotation, the alignments of predicted proteins against databases, including KEGG [20], COG [21], Swiss-Prot [22], TrEMBL [23] and nr (at NCBI, National Center for Biotechnology Information), were carried out with the program blastall (version 2.2.21) [24]. Genes encoding transfer RNA (tRNA) and their secondary structure were predicted using tRNAscan [25]. Genes encoding ribosomal RNA (rRNA) were predicted through RNAmmer [26] or homologous comparison. Genes encoding other RNA, including microRNA (miRNA), small RNA (sRNA) and small nuclear RNA (snRNA) were predicted by Rfam [27]. Transposons were identified through RepeatMasker and RepeatProteinMasker [28].

After the assembly, global guanine-cytosine composition of the whole non-redundant sequence was calculated with the in-house program (BGI, Beijing, China). The predicted open reading frames (ORFs) inferred by Glimmer were also counted for GC content and length distribution.

### Promoter and signal peptide prediction

The promoter, including -35, -10 and transcription start site (TSS) regions, were predicted through Neural Network Promoter Prediction [29] and Sequence Alignment Kernel [30] methods. The signal peptide for secreted proteins was predicted by SignalP [31].

### Multiple sequences alignments and identification of protein motifs

Multiple alignments of predicted proteins with conserved sequences were performed with Constraint-based Multiple Alignment Tool (COBALT) [32] at NCBI. Conserved motifs were identified by searching sequences against the COG database.

### Evolutionary and phylogenetic analysis

The distances between conserved sequences were calculated from the multiple alignments with ClustalW [33]. Neighbor-joining tree phylograms were constructed, bootstrapped (500 replications), and drawn in MEGA (version 5.03) [34].

With the evolutionary distances, the evolutionary pressures imposed on keeping any two genes in the genome were inspected by computing a Pearson correlation coefficient for any two genes. The missing genes (designated as "ND") were useful in inspecting the co-evolutionary relationships, and usually defined as zero. However, here, the smaller the evolutionary distance, the closer a homologue is to the gene in TD01. To make use of the missing genes, we transformed the evolutionary distances into similarity scores with e^(-1*distance) ^and defined the missing genes (ND) as zero, i.e. a shorter distance relates to an increased similarity score. Then a Pearson correlation coefficient was computed for any two genes using the converted similarity scores.

### Calculation of isoelectric point values

FASTA format protein sequences, either predicted from *Halomonas *sp. TD01 or retrieved from NCBI were submitted to ExPASy Proteomics server (http://web.expasy.org/compute_pi/). The isoelectric point (pI) distribution of each strain was counted with an interval of 0.2 differences from 3 to 13.

### Accession numbers

The draft genome assemblies for *Halomonas *sp.TD01 were submitted into the GenBank database; its accession number was GenBank: AFQW00000000. Other accession numbers of the sequences involved in this study are listed in Table 1 and Additional file 1, Table S1.

**Table 1:** Putative PHA and ectoine relevant genes in the genome of *Halomonas *sp. TD01

	Enzyme	Gene	Accession Number^a^	Calculated Molecular mass (kDa)^b^	Calculated pI^b^
PHA	Polyhydroxyalkanoate synthase	*phaC1*	EGP20415	70	4.80
		*phaC2*	EGP19504	92	5.99
	Polyhydroxyalkanoate depolymerase	*phaZ1*	EGP20509	40	7.75
		*phaZ2*	EGP18355	33	4.80
		*phaZ3*	EGP19590	38	4.68
	Phasin	*phaP*	EGP20414	16	5.64
	Polyhydroxyalkanoate synthesis repressor	*phaR*	EGP21321	18	4.78
Ectoine	L-2,4-diaminobutyric acid acetyltransferase	*ectA*	EGP18461	21	4.94
	L-2,4-diaminobutyric acid transaminase	*ectB*	EGP18460	46	5.69
	Ectoine synthase	*ectC*	EGP18459	15	5.03
	Ectoine hydroxylase	*ectD*	EGP18127	36	5.35

^a^, GenBank accession number.

^b^, Molecular mass (kDa) and pI were calculated through ExPASy Proteomics server (http://web.expasy.org/compute_pi/).

## Results

### Overview of sequencing and gene prediction

Sequencing was conducted with Solexa technology. Using SOAPdenovo software, the clean raw data was assembled into 26 scaffolds varying from 511 to 918,836 bp with a total length of 4,092,837 bp. The estimated percentages of genome coverage were 102.64% and 99.56% based on k-mer analysis and reads comparison, respectively. 3,894 Open Reading Frames (ORF) predicted by Glimmer had occupied 89.18% of the whole assembled sequences. The GC content within coding sequences was 53.23%, which was a little higher than that of the whole sequences (52.57%). Most putative proteins were distributed within the length ranges between 200 and 300 amino acids (aa) (Additional file 2, Figure S1). In addition, the putative proteins were classified according to the COG function (Additional file 3, Figure S2A) and KEGG pathway (Additional file 3, Figure S2B). The comparison of the predicted protein sequence set of *Halomonas *sp. TD01 with the nr database (NCBI) revealed its close relationship with *Halomonas elongata *DSM 2581, *Chromohalobacter salexigens *DSM 3043 and *Aromatoleum aromaticum *EbN1. Although the sequence of the scaffolds of *Halomonas *sp. TD01 have not been confirmed, the alignment between the chromosome of *C. salexigens *and the scaffolds of *Halomonas *sp. TD01 showed distinguishable co-linearity (Additional file 4, Figure S3).

### Identification and evolutionary analysis of PHA relevant genes from *Halomonas *sp. TD01

The genes relevant to PHA were identified through homologous alignments against the public annotation databases, including KEGG, COG, Swiss-Prot, TrEMBL and nr, using the BLAST program. As inferred from the similarity results, there might be two putative genes encoding PHA synthases (PhaC), three encoding PHA depolymerases (PhaZ), one encoding phasin (PhaP) and one encoding PHA synthesis repressor/regulator (PhaR, which showed 45% identities with YP_725943 of *Ralstonia eutropha *H16) in the genome of halophilic PHBV producing strain *Halomonas *sp. TD01 (Table 1). In addition, although the putative PhaC1 shared only 19% identity with PhaC2, both of them hit COG (clusters of orthologous groups) 3243 in the conserved domain database (CDD) at NCBI, implying that they belonged to PHA synthases [35]. Similarly, all the three PhaZs deduced from the genome of *Halomonas *sp. TD01 shared remarkably the same hits on COG3509 and pfam10503 in CDD, which strongly proposed their functions as PHB depolymerases. Phylogenetic trees clearly illustrated that the putative PhaZ, PhaP and PhaR from *Halomonas *sp. TD01 had close relationships with the corresponding, well-characterized enzymes from non-halophiles, which presented clear, *in silico *evidence for their function in PHA degradation and regulation (not shown).

**Figure 1:**
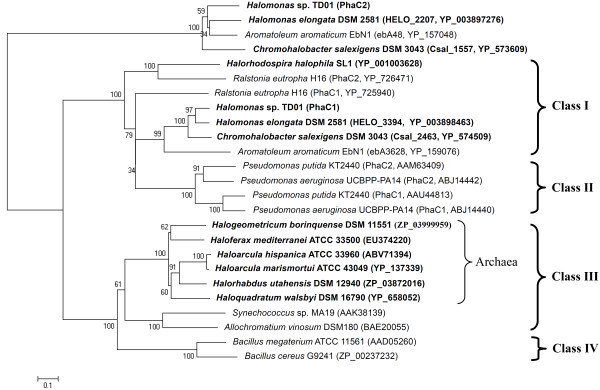
**Phylogenetic tree of putative PhaC from *Halomonas *sp. TD01 with reported PHA synthases**. Halophiles were highlighted in bold. The trees were constructed using the neighbor-joining algorithm with MEGA (version 5.03) software. The GenBank accession numbers were given after the microorganism names. The numbers besides the nodes indicated the bootstrap values based on 500 replications. Bar 0.1 substitutions per site were indicated on the graph.

**Figure 2:**
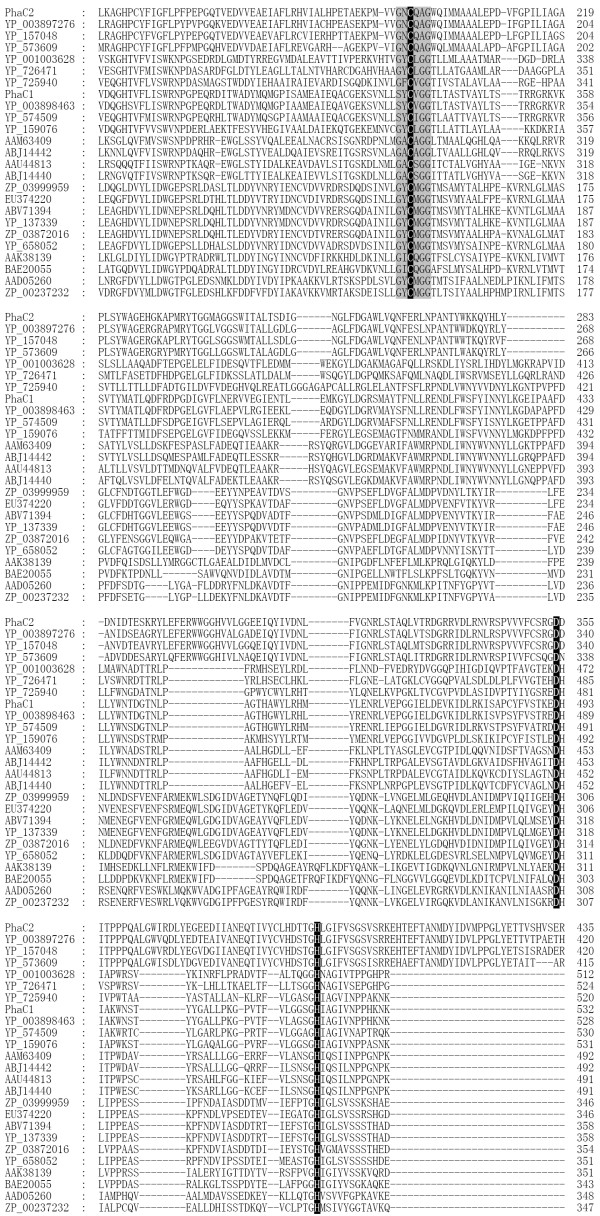
**Multiple alignment of putative PhaC from *Halomonas *sp. TD01 with reported PHA synthases**. Black shading indicated the conserved catalytic triad residues, and gray shading indicated lipase-like box residues. The sequences were listed in the same order of the phylogenetic tree in Figure 1.

The evolutionary differences of PHA synthases between archaeal-, bacterial- and non-halophiles were revealed by phylogenetic analysis (Figure 1). PHA synthases are clustered into different groups (Figure 1). The PhaCs from haloarchaea shared general features with class III enzymes, forming a novel subclass; the PhaCs from halophilic bacteria were closer to class I enzymes [15]. PHA synthases of class I and II appeared to originate from a common ancestor, while synthases of class III and IV originated from an alternative common ancestor (Figure 1). However, the common ancestor of putative PhaC2 from strain TD01 and its homologues seemed relatively distant from the well-characterized PHA synthases in the phylogenetic analysis (Figure 1). Furthermore, the molecular masses of putative PhaC2 and its homologues were between 81 and 92 kDa, while the Class I enzymes are usually between 61 and 73 kDa [1]. Revealed by the multiple alignments, the putative PhaC2 from *Halomonas *sp. TD01 shared longer C-terminus with those homologues from *H. elongata *DSM 2581, *C. salexigens *DSM 3043 and *A. aromaticum *EbN1 than that of the well-studied classical class I PHA synthases (Figure 2). It is possible that the longer C-terminus of the PhaC2 was important for the function of PHA synthase at high salt concentration, which was observed in the haloarchaeal PhaC [15,16]. However, their N-terminus, which proved quite important for the function of classical PHA synthases [36,37], was shorter than that of the well-studied classical class I PHA synthases (Figure 2). Yet, enzymatic and structural studies are required to determine whether the putative PhaC2 from *Halomonas *sp. TD01 and its homologues are a novel subclass of PHA synthases. Although there is still uncertainty about the classification of PhaC2 from *Halomonas *sp. TD01, PhaC1 from the strain TD01 exhibited relatively high similarities with the classical class I PHA synthases. For example, PhaC1 from TD01 has high sequence similarity (44% and 34% sequence identities with YP_725940 and YP_726471, respectively) with the known class I PHA synthases from *Ralstonia eutropha *H16 (Figure 1). The molecular mass of PhaC1 was about 70 kDa, which is within the common range of well-studied class I enzymes [1]. In addition, the conserved lipase-like box residues were recognized in the PhaC1 enzyme (Figure 2).

The detailed analysis through multiple sequences alignments of putative PhaC1 and PhaC2 with their homologues was performed. The most similar homologues of both PhaC1 and PhaC2 came from *H. elongata *DSM 2581 (78%, 73% identities, respectively), *C. salexigens *DSM 3043 (67%, 67% identities, respectively) and *A. aromaticum *EbN1 (48%, 69% identities, respectively) (Additional file 5, Table S2). The multiple sequences alignments showed strong conservation between these protein sequences and the well-characterized PHA synthases (Figure 2). The conserved lipase-like box residues were recognized in all of these proteins (Figure 2). In addition, three residues (cysteine, aspartic acid and histidine), composing the conserved catalytic triad of PHA synthases, were also identified in these proteins (Figure 2). However, it is interesting to find that, instead of the traditional lipase-box pattern "GxCxG", all PhaC1 homologues (listed in Figure 2, except for YP_159076 from non-halophilic *A. aromaticum *EbN1), have "SxCxG", and all the PhaC2 homologues (listed in Figure 2) have "GxCxA". These interesting patterns may imply the existence of a new class of PHA polymerases among the halophilic bacteria. Yet, more experimental data are needed to elucidate whether these patterns are related to the hypersaline environments.

With the progress on the research of PhaCs from halophiles, our vision on PHA synthesis in these strains was amazingly broadened [38]. In contrast, few efforts had been devoted to the studies on other enzymes involved in PHA metabolism excluding PhaC. The putative PhaZ3 was supposed to be a secreted protein through the prediction of signal peptides (Additional file 6, Figure S4), whereas the other two PHA depolymerases (PhaZ1 and PhaZ2) lack signal peptides and may be responsible for the intracellular PHA degradation.

**Figure 3:**
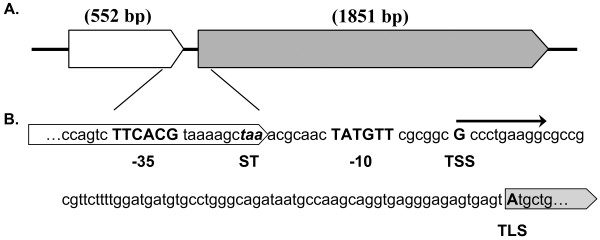
**The organization of *phaP *and *phaC1***. (A) The phasin gene (*phaP*) was located on the upstream of *phaC1 *with a space of 92 bp. (B) The predicted promoter structure of *phaC1*. Bold capital letters indicated key base pairs. TSS, transcription start site; TLS, translation start site of *phaC1*; ST, stop codon of *phaP*; -10, Pribnow box; -35, -35 element. Arrow indicated the transcription from TSS.

Most of PHA relevant genes are scattered in the genome, except for the *phaP *and *phaC1*, which were connected with a space of 92 bp (Figure 3A). However, an independent promoter of *phaC1*, with highly conserved -35 and -10 binding sites for σ^70^, was identified (Figure 3B). The -35 motif extended into the tail of *phaP *coding region, and the putative transcription start site (TSS) was 73 bp on the upstream of the translation start site (TLS) of *phaC1 *gene (Figure 3B). The existence of an independent promoter of *phaC1 *suggested the separated regulation on the gene expression of *phaP *and *phaC1*. It was understandable that phasins were usually expressed in large quantities during PHA storage period, whereas massive expression of PHA synthases was required during synthesis period [39,40].

### Osmoregulatory mechanisms of *Halomonas *sp. TD01 inferred from the genome information

The pI values provide simple indication of protein acidity. To compare the differences of protein acidity between halophiles and non-halophiles, the pI distribution of representative proteome from halophilic bacterium *Halomonas *sp. TD01 and *Halomonas elongata *DSM 2581, halophilic arhaeon *Haloarcula marismortui *ATCC 43049 and non-halophilic *E. coli *MG1655, was calculated with the interval of 0.2 pI from 3 to 13. Bimodal distribution of pI appears clearly on the plot for each strain (Additional file 7, Figure S5). Both acidic peaks of *Halomonas *sp. TD01 and *H. elongata *DSM 2581 were at 5.1, which were a little lower than the non-halophilic *E. coli *MG1655 (at 5.5), but much higher than the extremely halophilic archaeon *H. marismortui *(at 4.3). While *H. marismortui *only showed a small peak representing basic proteins, the other three strains shared the distinct basic peaks at around pI 9.5. The median pI values of *Halomonas *sp. TD01, *H. elongata*, *H. marismortui *and *E. coli *were 5.67, 5.51, 4.46 and 6.17, respectively, which displayed a similar pattern with the pI distribution.

**Figure 4:**
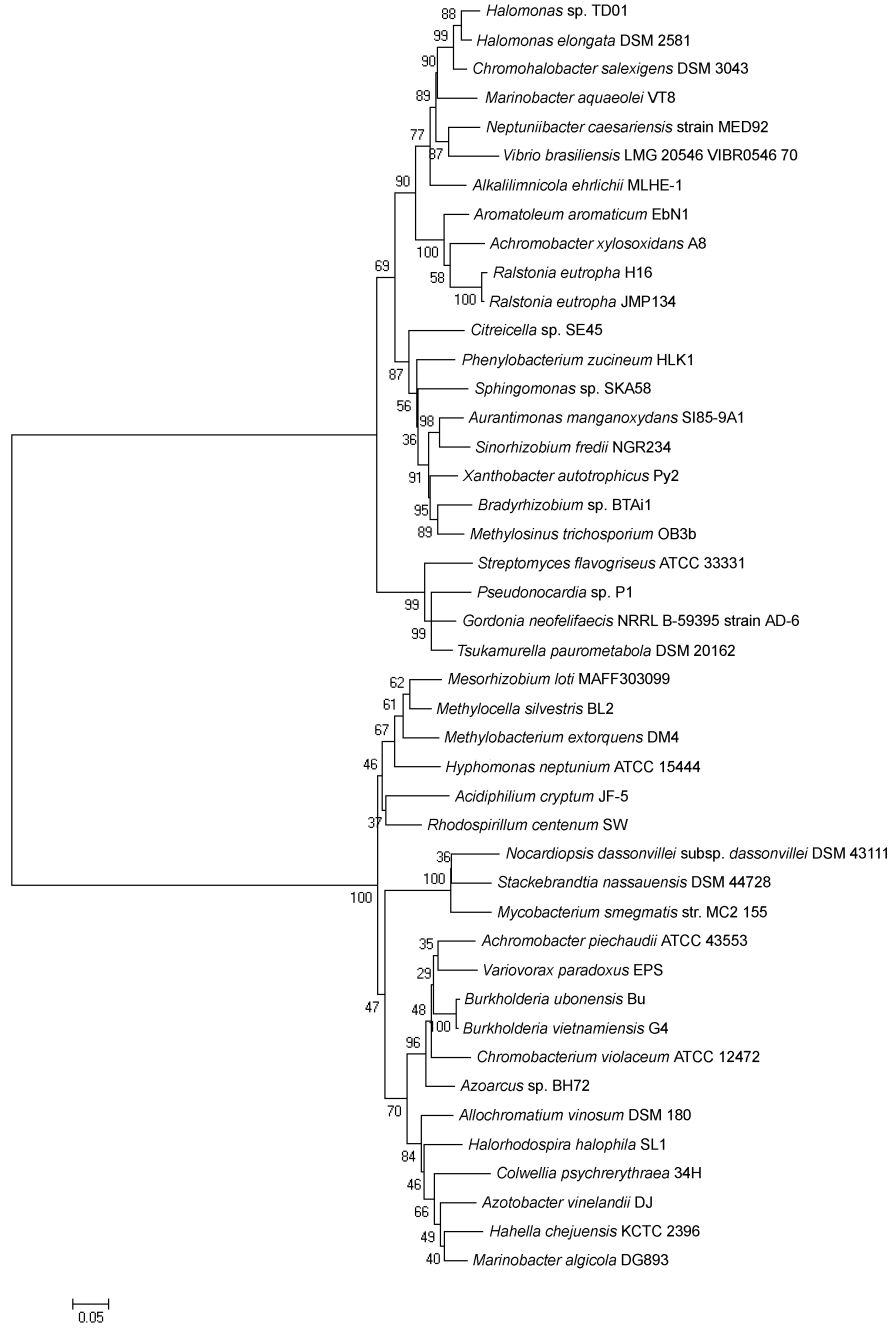
**Phylogenetic tree based on the 16S rDNA sequences of strains with homologues of PHA and ectoine relevant enzymes**. The trees were constructed using the neighbor-joining algorithm with MEGA (version 5.03) software. The numbers besides the nodes indicated the bootstrap values based on 500 replications. Bar 0.05 substitutions per site were indicated on the graph. The GenBank accession numbers of 16S rDNA sequences of the strains with the homologues of PHA and ectoine relevant enzymes were listed in Additional file 1, Table S1.

As moderate halophiles, accumulation of organic osmolytes, instead of inorganic ions was supposed to be the main strategy to deal with high osmotic environment [8]. So far, many organic solutes, characterized with small molecule masses and fast turnover through *de novo *synthesis or absorption from environment and degradation, were identified to function as osmoregulators [41]. The genes relevant to betaine and ectoine, two of the most widespread compatible solutes in nature, were identified and annotated based on the genome information of *Halomonas *sp. TD01. There were two putative genes clusters encoding compatible solute synthesis systems for betaine (*betABI*) and ectoine (*ectABC*) (Table 1 and Additional file 8, Table S3). The genes (*doeABCD*) involved in the degradation of compatible solutes were also discovered in the genome of *Halomonas *sp. TD01 (Additional file 8, Table S3) [41]. In addition, five genes encoding the ATP-binding cassette (ABC)-type proline/glycine betaine transporters (ProU) and eight genes encoding betaine/carnitine/choline transporters (BCCT family) were identified through the comparison with well-studied betaine transporters (Additional file 8, Table S3) [42]. The ABC-type transporters generally showed broader substrate specificities than the ones belonging to the BCCT family [43]. In contrast, only one gene (*teaA*) encoding a homologue of tripartite ATP-independent periplasmic transporters (TRAP-T) for ectoine was unveiled (Additional file 8, Table S3) [44]. Most of these osmolytes relevant genes shared close similarities with the homologues from *C. salexigens *and *H. elongata*, as revealed by evolutionary analysis (Additional file 5, Table S2). These similarities were also observed in their PHA relevant genes (Additional file 9, Figure S6) and 16S rDNA sequences (Figure 4) based phylogenetic analysis.

### The evolutionary analysis of PHA and ectoine relevant proteins from *Halomonas *sp. TD01

As revealed through the analysis of the osmoregulatory mechanism, *Halomonas *sp. TD01 was possibly able to synthesize ectoines with commercial interest as protectants against proteolysis [18]. The existence of PHA and ectoine synthesis genes qualifies *Halomonas *sp. TD01 as a candidate for the combined production of PHA and osmolytes [7]. Moreover, it was interesting to observe that many species other than halophiles possessed both homologues of putative PHA and ectoine relevant enzymes from *Halomonas *sp. TD01 (Additional file 1, Table S1). Based on the evolutionary distances of 16S rDNA (Figure 4), PHA and ectoine relevant protein sequences, *Halomonas *sp. TD01 shared closest relationship with *Chromohalobacter salexigens *DSM 3043 and *Halomonas elongata *DSM 2581, which were also known as halophilic bacteria and belonged to the same family *Halomonadaceae *as the strain TD01 (Additional file 5, Table S2). It was interesting to find that, with regard to PHA relevant enzymes, similarities (the average evolutionary distance of PhaCs: 0.666) between TD01 and rhizobia strains, which fix nitrogen and are symbiotic with plant roots, were significant (Additional file 5, Table S2). The homologues of ectoine synthesis and PHA degradation (PhaZs) enzymes from *Halomonas *sp. TD01 were present in some species belonging to the class *Actinobacteria*, are a group of Gram-positive bacteria with high G+C content (Additional file 5, Table S2). In addition, it was noticed that only proteins from some *γ-proteobacteria *species exhibited significant similarity with the PhaP from strain TD01 (Additional file 5, Table S2), which implies this PhaP possibly possesses a relative independent evolution pathway in the *γ-proteobacteria*. However, it seemed that the other homologues of strain TD01 spread in *α-*, *β-*, *γ-proteobacteria *(Additional file 5, Table S2), suggesting that PHA and ectoine related enzymes from the halophilic strain TD01 shared common ancestors with the non-halophiles.

**Figure 5:**
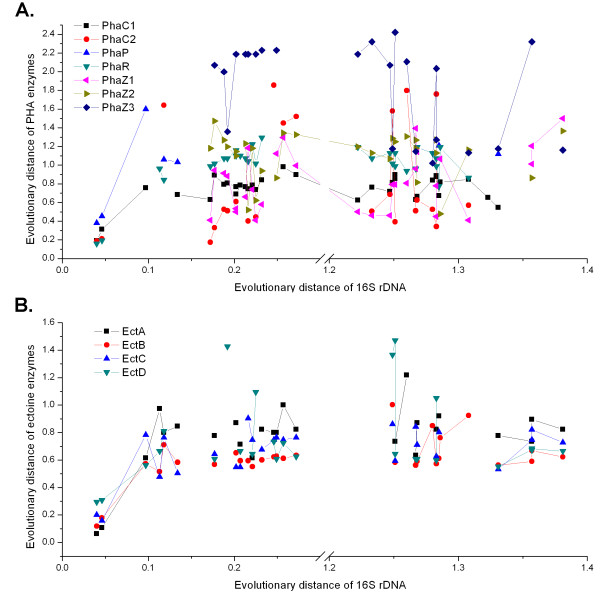
**Evolutionary analysis based on the 16S rDNA, PHA and ectoine relevant enzymes sequences**. The evolutionary distances of 16s rDNA, PHA (A) and ectoine (B) enzymes between *Halomonas *sp. TD01 and other strains were calculated from the multiple alignments with ClustalW [33]. For each strain, the evolutionary distance of 16s rDNA was plotted on the X axis, and evolutionary distances of PHA (A) and ectoine (B) relevant enzymes were plotted on the Y axis. The same enzyme of different species was linked, while broken lines indicated the genes missing from the genomes of the corresponding strains (Additional file 1, Table S1).

The plots of evolutionary distances of PHA and ectoine relevant enzymes versus that of 16S rDNA were drawn with the aim of better understanding of the evolutionary relationship among these genes and 16S rDNA (Figure 5). When the evolutionary distances of 16S rDNA were lower than 0.15, there was an obvious co-linearity between the evolutionary distances of 16S rDNA and those of the ectoine relevant enzymes (Figure 5B). Yet, among the PHA relevant enzymes, only the evolutionary distances of PhaC1 and PhaP showed some co-linearity with those of 16S rDNA when the evolutionary distances of 16S rDNA were lower than 0.15 (Figure 5A). However, when the evolutionary distances of 16S rDNA were higher than 0.15, the evolutionary distances of PHA and ectoine relevant enzymes seemed non-colinear with those of 16S rDNA (Figure 5). Inferred from this non-colinearity, there may be extensive horizontal gene transfer of PHA and ectoine relevant genes between the ancestors of these species listed in Additional file 1, Table S1. For example, although *Azotobacter vinelandii *DJ was evolutionarily distant from strain TD01, their *phaC *and *phaR *genes shared significant similarities; the *phaR *genes of *Achromobacter piechaudii *ATCC 43553, which was distantly related to TD01 with respect to 16S rDNA sequences, shared significant similarities with that of TD01 (Additional file 5, Table S2). Although PhaC, PhaP, PhaR, and PhaZ genes were all involved in PHA metabolisms, they were under different horizontal gene transfer (Figure 5A). The fact that there were similar evolutionary distances of ectoine genes from closely or distantly related genomes to *Halomonas *sp. TD01 (Figure 5B), may suggest that there were also horizontal gene transfer of ectoine genes. *Mycobacterium smegmatis *str. MC2 155 and *Stackebrandtia nassauensis *DSM 44728, for instance, were distantly related to strain TD01 based on 16S rDNA sequences, they shared similar ectoine genes with that of *Halomonas *sp. TD01 (Additional file 5, Table S2). Moreover, there were some similarities between the phylogenetic trees of 16S rDNA (Figure 4) and PHA, ectoine relevant enzymes (Additional file 9, Figure S6), especially for the closely related strains *Halomonas elongata *DSM 2581, *Chromohalobacter salexigens *DSM 3043. Although the sequence similarity of PHA and ectoine relevant genes implies the existence of horizontal gene transfer events between different species, no distinguishable transposable elements, which were identified through the RepeatMasker and RepeatProteinMasker [28], were found flanking these genes in the genome of *Halomonas *sp. TD01 (Additional file 10, Table S4 and S5). There remained a challenge to disclose how these genes transferred between the different species.

**Figure 6:**
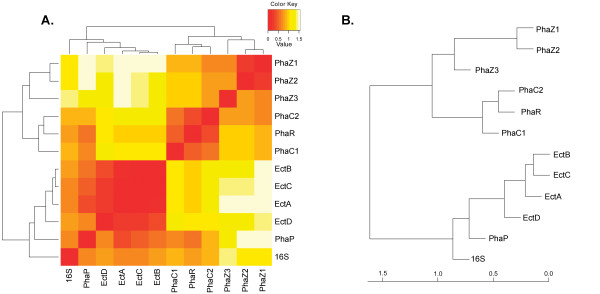
**The correlation of evolutionary distance for any two of the 16S rDNA, PHA and ectoine relevant sequences**. The heatmap (A) and cluster dendrogram (B) based on their evolutionary correlation coefficient. The self-distance was set at zero. Then, the heatmap was plotted by using the function heatmap.2 in package gplots of R programming language. The color intensity in the heatmap corresponded to the distance. The cluster dendrogram was plotted by the hierarchical clustering method in R programming language.

To further understand the functional relationships between PHA and ectoine relevant enzymes, Pearson correlation coefficient was calculated after the transformation of evolutionary distances, with the aim of illustrating the evolutionary pressure imposed to keep any two genes in the genome. It was obvious that EctA, EctB, EctC, and EctD were highly related to each other, as they almost co-occur in the genome of all strains investigated in this study, and the correlations of their evolutionary distances to those in TD01 were very high (Figure 6). This was in agreement with the fact that they formed an operon in the genome of some strains, such as *Sphingomonas *sp. SKA58 and *Stackebrandtia nassauensis *DSM 44728, etc (Additional file 1, Table S1). In addition, as elucidated in the heatmap and cluster dendrogram, PhaZ1, PhaZ2 and PhaZ3 were closely related to each other, and all of them share distinct relationship with other PHA genes (Figure 6). Within these three PHA depolymerases, PhaZ1 and PhaZ2 were more closely related to each other than to PhaZ3, which was in accordance to the fact that PhaZ1 and PhaZ2 were putative intracellular depolymerases, whereas PhaZ3 was putative extracellular depolymerase, as suggested by the signal peptide prediction (Additional file 6, Figure S4). Although PhaP seemed evolutionarily closer to ectoine relevant enzymes, this was probably because it was absent in 36 out of 44 strains (Additional file 1, Table S1). More studies including both *in silico *and wet-experiment, should be implemented for further clarification of their relationships. As illustrated by Figure 6, it seemed that there were not high evolutionary pressures to keep PHA genes together in the same genome, and whether to keep or lose certain PHA genes may be dependent on specific environment of each strain. For instance, *Ralstonia eutropha *H16, a ubiquitous inhabitant of soil and freshwater, suffers most from energy limitation, and has been well adapted with the accumulation of high content PHA as carbon reservoir. Therefore, multiple PHA relevant genes were maintained in its genome, whereas ectoine genes were missing as there was no environmental pressure for the presence of ectoines. This suggested that genetic engineering of single PHA related genes, such as knockout or addition of PHA genes, may not have astonishing impacts on bacterial growth and PHA production, yet it may be necessary in order to achieve higher PHA production in some strains, such as *Halomonas *sp. TD01. In contrast, the evolutionary pressures to keep ectoine genes together in the same genome were much higher (Figure 6), suggesting these genes may all be necessary for ectoine metabolism. Thus, inhibiting the expression of any of the four ectoine genes may have a strong impact on the ectoine biosynthesis and the survival in high osmotic environments. This finding may help develop better genetic engineering strategies for future production of PHA and ectoine.

## Discussion

So far, there have been relatively few studies on the PHA synthases from halophilic microorganisms [7]. Recently, the detailed studies on the PHA synthases from the halophilic archaea have extended our insights on their classification, providing solid evidences to support the existence of a novel subclass (IIIA) synthases with distinguished features from the subclass (IIIB) isolated from bacteria [15]. The widespread novel PHA synthases in halophilic archaea consist of two subunits, PhaC and PhaE, both of which are required for their activities [38]. However, these novel PHA enzymes are clustered separately from the ones isolated from the halophilic bacteria, including *Halomonas *sp. TD01, *H. elongata*, *C. salexigens*, and *Halorhodospira halophila*, etc (Figure 1). As illustrated by the phylogenetic analysis, PHA synthases (especially the putative PhaC1) from halophilic bacteria, shared the significant similarities with class I enzymes, which were different from the well-studied enzymes from halophilic archaea (Figure 1) [7]. PhaCs in *Halomonas *sp.TD01 were able to synthesize PHB with a molecular weight of 600 kDa in fed-batch fermentation, which was in accordance with the characteristics of class I PHA synthases [1,13]. The conserved catalytic residues, firstly discovered in the PHA synthases of *Chromatium vinosum *and *R. eutropha*, were also recognized in PhaC1 and PhaC2 from *Halomonas *sp. TD01 (Figure 2), indicating that they share similar catalytic mechanisms and substrate specificities [7,45]. In addition, PHBV was detected when glucose and propionic acid (or valeric acid) were supplemented as carbon sources in the medium, which also agreed with the features of class I synthases [13]. Under the catalysis of putative PhaC1 and/or PhaC2, PHBV accumulation was able to reach 80 wt% of cell dry weight consisting of 30% 3-hydroxyvalerate 3HV fraction when supplemented with valeric acid as additional carbon source, which qualified *Halomonas *sp. TD01 as a strain of industrial interest for PHBV production [13]. Nevertheless, physiological and enzymatic characterization of the PHA synthases from halophilic bacteria are still required to elucidate whether the putative PhaC2 from *Halomonas *sp. TD01 and its homologues form their own class of PHA synthases, as implied by the phylogenetic studies and multiple sequences alignment of putative PhaC2 (Figure 2).

While PHA synthases from halophiles have received attentions, the studies on PHA depolymerase, regulator/repressor and phasin from halophiles, which also play crucial roles in the PHA cycle, remained unclear [15]. Evolutionary analysis demonstrated that the PhaC, PhaZ, PhaP, PhaR and ectoine relevant enzymes from halophilic *Halomonas *sp. TD01 share close relationships (the average evolutionary distance: 0.945) with those from non-halophilic strains (Additional file 5, Table S2). It is very clear that further systematic and detailed studies on these genes from halophiles would improve our understanding on their functions and evolution, potentially leading to enhancing PHA production at reduced costs. Moreover, genome-scale metabolic analysis would also advance the progress of PHA production by halophiles [46].

The most well-known genetic organization of representative PHA synthases in class I was the *phbCAB *operon from *R. eutropha*, containing genes encoding PHA synthases, acetyl-CoA acetyltransferase and 3-hydroxybutyryl-CoA dehydrogenase in sequence [1]. However, in *Halomonas *sp. TD01, *phaC *and *phaA*, *phaB *genes were scattered throughout the genome (Table 1, Additional file 8, Table S3). More interestingly, the neighborhood genetic organization of the homologues of putative *phaP *and *phaC1 *from *Halomonas *sp. TD01 was also conserved in both halophiles and non-halophiles exclusively from *γ-proteobacteria*, which can imply that there may be extensive horizontal gene transfers between their ancestral strains, possibly suggesting a same living environment in the past (Table 2). The interval lengths between *phaC1 *and *phaP *genes varied from 55 to 204 bp (Table 2). Most of these species were isolated from an aquatic (especially oceanic) environment, similar to the saline lake from where *Halomonas *sp. TD01 was isolated [13]. It is possible that the horizontal gene transfer may have occurred in ancient times, when the universal common ancestors of these *γ-proteobacteria *mentioned in Table 2 survived in the same aquatic environment, leading to the acquisition of adjacent *phaP *and *phaC1 *genes by the ancestor of *Halomonas *sp. TD01. With the geological evolution, the ancestors of these *γ-proteobacteria *were then separated into their present environments and the *phaC1 *and *phaP *genes with their interval sequences evolved independently to their current status. The conservation of adjacent organization could play an important role in the regulation and metabolism of PHA cycles in *γ-proteobacteria*, which deserves further study.

**Table 2:** The intervals lengths between the co-linear *phaP *and *phaC1 *genes from *Halomonas *sp. TD01 and their homologues in other microorganisms

*Order*	*Family*	*Species*	*Interval (bp)*
*Oceanospirillales*	*Halomonadaceae*	*Halomonas *sp.TD01	92
		*Halomonas elongata *DSM 2581	82
		*Chromohalobacter salexigens *DSM 3043	204
	*Oceanospirillaceae*	*Marinomonas *sp. MED121	92
	*Hahellaceae*	*Hahella chejuensis *KCTC 2396	134
*Vibrionales*	*Vibrionaceae*	*Vibrio alginolyticus *12G01	66
		*Vibrio brasiliensis *LMG 20546	55
		*Vibrio caribbenthicus *ATCC BAA-2122	57
		*Vibrio cholerae *1587	57
		*Vibrio coralliilyticus *ATCC BAA-450	58
		*Vibrio furnissii *CIP 102972	56
		*Vibrio harveyi *1DA3	66
		*Vibrio metschnikovii *CIP 69.14	116
		*Vibrio mimicus *VM573	57
		*Vibrio orientalis *CIP 102891	57
		*Vibrio parahaemolyticus *RIMD 2210633	66
		*Vibrio shilonii *AK1	79
		*Vibrio sinaloensis *DSM 21326	58
		*Vibrio *sp. Ex25	80
		*Vibrio *sp. RC586	57
		*Vibrio splendidus *LGP32	73
		*Vibrio vulnificus *CMCP6	61
		*Vibrionales bacterium *SWAT-3	75
		*Grimontia hollisae *CIP 101886	63
		*Photobacterium angustum *S14	211
		*Photobacterium damselae *subsp. *damselae *CIP 102761	59
		*Photobacterium leiognathi *subsp. *mandapamensis svers*.1.1.	119
		*Photobacterium profundum *3TCK	179
		*Photobacterium profundum *SS9	65
		*Photobacterium *sp. SKA34	121
*Pseudomonadales*	*Moraxellaceae*	*Enhydrobacter aerosaccus *SK60	199
*Alteromonadales*	*Shewanellaceae*	*Shewanella halifaxensis *HAW-EB4	94
		*Shewanella pealeana *ATCC 700345	95
	*Alteromonadaceae*	*Marinobacter aquaeolei *VT8	74
		*Marinobacter *sp. ELB17	118
*Aeromonadales*	*Aeromonadaceae*	*Aeromonas caviae*	92
		*Aeromonas hydrophila*	92
		*Aeromonas hydrophila *subsp. *hydrophila *ATCC 7966	93
		*Aeromonas salmonicid*a subsp. *salmonicida *A449	93

Wild-type cells require PHA synthesis and degradation cycle to maintain carbon supply balance under changing environments [2]. However, for industrial production of PHA, degradation is not favored, because it leads to the waste of carbon sources and consequently reduces PHA accumulation [47,48]. For example, it has been reported that the knockout of *phaZ *in *P. putida *KT2442 inhibited the decrease of PHA content during batch fermentation processes [47]. For *Halomonas *sp. TD01, the presence of three putative PHA depolymerases likely decreased PHA accumulation in batch fermentation processes (unpublished observations). Thus, identification of these *phaZ *genes could help the intentional knockout of PHA degradation pathways in *Halomonas *sp TD01, in the hope of promoting more PHA accumulation. In addition, distinguishing the intracellular (PhaZ1 and PhaZ2) and extracellular (PhaZ3) depolymerases allow the possibility to identify targets of most importance, reducing the time and labor required for gene knockout.

To maintain the balance of osmotic pressure across the cell membrane, high concentrations of ions or organic osmolytes are accumulated in the cells [49]. Neutral proteins would become denatured in hypersaline condition, while acidic ones, with more negatively charged amino acid residues on the surfaces, were able to stay functional through the binding of hydrated salt ions in the cytoplasm [50]. It was reported that the proteomes of extremely halophilic archaea, such as *H. marismortui*, showed a notable general tendency towards acidity, which was illustrated by the pI distribution (Additional file 7, Figure S5) [49]. Contrary to this, less acidic proteomes from halophilic bacteria, including *Halomonas *sp. TD01 and *H. elongata*, were observed and implied their decreased dependence on the absorption of salt to maintain ionic balance in hypersaline environments [51]. To survive in hypersaline conditions, it was believed that halophilic *Halomonas *sp. TD01 adopted another universal strategy, accumulating organic compatible solutes instead of inorganic ions to balance the osmotic pressure with a surprisingly broad salt concentration range [8]. This was illustrated by the widespread of biosynthesis and transporter genes for multiple organic osmolytes over the genome. Even though halophilic microorganisms seemed to optimize their metabolism to minimize the energetic cost for osmotic adaptation, the *de novo *biosynthesis of osmolytes was less favorable with respect to either energy consumption or flexibility than their absorption from environment [52]. In addition, there are many transporters for betaine, which possibly suggested that betaine was also one of the compatible solutes utilized by *Halomonas *sp. TD01 [53]. However, a complete comprehension of the occurrence and distribution of organic osmoregulatory solutes in *Halomonas *sp. TD01 remain to be determined. Moreover, as the environmental factors, especially the nutritional ones, are able to strongly influence the production of PHA, the effects of both environmental factors and genetic background of *Halomonas *sp. TD01 on the production of PHA should be further studied.

## Conclusions

In conclusion, the disclosure of the genome sequences of *Halomonas *sp. TD01 improves our understanding on the metabolism and evolutionary relationship of PHA and osmoregulatory solutes from halophilic bacteria. Detailed and systematic *in silico *analysis on the PHA and osmolytes relevant genes provides abundant insights on their classifications, functions and phylogeny. Osmoregulatory mechanisms were also discussed through the comparison of pI distribution. The availability of genomic information would inevitably pave new ways for the application of numerous post-genomic technologies and accelerate our work in the genetic optimization of *Halomonas *sp. TD01 for the industrial production of PHA, and possibly accompanied with the co-production of compatible solute ectoine.

## Competing interests

The authors declare that they have no competing interests.

## Authors' contributions

LC designed, performed most parts of the study, and prepared the manuscript. DT designed and performed sequencing of genome. GA and JCC participated in the screening of the strain and the sequencing of the genome. XRD performed the Pearson correlation study. WDT designed the evolutionary distance study and revised the manuscript. GQC designed and supervised the experiments, and revised the manuscript. All authors read and approved the final manuscript.

## Supplementary Material

Additional file 1 **Table S1**. Accession numbers of 16S rDNA, putative PHA and ectoine relevant proteins from *Halomonas *sp. TD01 and other strains. Click here for file

Additional file 2 **Figure S1**. Gene length distribution of *Halomonas *sp. TD1. Click here for file

Additional file 3 **Figure S2**. COG function and KEGG pathway classification of *Halomonas *sp. TD1. Click here for file

Additional file 4 **Figure S3**. Alignment of the chromosomes of *C. salexigens *and *Halomonas *sp. TD1. Click here for file

Additional file 5 **Table S2**. Evolutionary distances of 16S rDNA, PHA and ectoine relevant proteins between *Halomonas *sp. TD01 and other species. Click here for file

Additional file 6 **Figure S4**. Signal peptide prediction of PhaZ3 with neural networks (NN) and hidden markov models (HMM). Click here for file

Additional file 7 **Figure S5**. Calculated isoelectric point (pI) distribution. Isoelectric point (pI) distribution of halophilic bacteria (*Halomonas *sp. TD01 and *Halomonas elongata *DSM 2581), haloarchaea (*Haloarcula marismortui *ATCC 43049) and non-halophilic bacterium (*Escherichia coli *MG1655) versus percentage of total proteins. Distribution with the interval of 0.2 pI was counted and plotted. Click here for file

Additional file 8 **Table S3**. Accession numbers of putative PhaA, PhaB and osmolytes relevant enzymes in the genome of *Halomonas *sp. TD01. Click here for file

Additional file 9 **Figure S6**. Phylogenetic trees based on the PhaC1 (A), PhaC2 (B), PhaP (C), PhaR (D), PhaZ1 (E), PhaZ2 (F), PhaZ3 (G), EctA (H), EctB (I), EctC (J) and EctD (K) sequences of *Halomonas *sp. TD01 with their homologues of other strains. Click here for file

Additional file 10 **Table S4 and S5**. Locus of putative PHA and osmolytes relevant genes in the genome of *Halomonas *sp. TD01; Transposon and TEprotein identification through the RepeatMasker and RepeatProteinMasker. Click here for file
