# Supplementary material for: Comparative genomics study of polyhydroxyalkanoates (PHA) and ectoine relevant genes from Halomonas sp. TD01 revealed extensive horizontal gene transfer events and co-evolutionary relationships
Source: Microb Cell Fact. 2011 Nov 1;10:88. doi: 10.1186/1475-2859-10-88 (PMC3227634; doi:10.1186/1475-2859-10-88)
Supplement: Additional file 1 — Table S1. Accession numbers of 16S rDNA, putative PHA and ectoine relevant proteins from Halomonas sp. TD01 and other strains. [file 1475-2859-10-88-S1.DOC]

**Table S1.** Accession numbers of 16S rDNA, putative PHA and ectoine relevant proteins from *Halomonas* sp. TD01 and other strains

| Species | 16S rDNA | PhaC1 | PhaC2 | PhaP | PhaR | PhaZ1 | PhaZ2 | PhaZ3 | EctA | EctB | EctC | EctD |
| --- | --- | --- | --- | --- | --- | --- | --- | --- | --- | --- | --- | --- |
| *Achromobacter piechaudii* ATCC 43553 | gb|ADMS01000149|:48-1578 | ZP_06686546 | ZP_06689470 | ND | ZP_06686548 | ZP_06688330 | ZP_06688330 | ZP_06688740 | ZP_06686912 | ZP_06686913 | ZP_06686914 | ZP_06686915 |
| *Achromobacter xylosoxidans* A8 | gi|311103224:2206254-2207784 | YP_003977718 | YP_003981131 | ND | YP_003977720 | YP_003980372 | YP_003980372 | YP_003980372 | YP_003978183 | YP_003978184 | YP_003978185 | YP_003978186 |
| *Acidiphilium cryptum* JF-5 | gi|148259021:2589571-2591063 | YP_001234431 | YP_001219969 | ND | YP_001235366 | YP_001219913 | YP_001219913 | ND | YP_001236117 | YP_001236118 | YP_001236119 | YP_001236120 |
| *Alkalilimnicola ehrlichii* MLHE-1 | gi|114319166:368208-369739 | YP_743071 | YP_743315 | YP_741176 | YP_743317 | ND | ND | ND | YP_742031 | YP_742030 | YP_742029 | YP_741236 |
| *Allochromatium vinosum* DSM 180 | gi|288939764:2906194-2907709 | ND | AAA23320 | ND | YP_003442068 | YP_003442374 | YP_003442374 | YP_003442374 | YP_003444769 | ND | ND | ND |
| *Aromatoleum aromaticum* EbN1 | gi|56475432:2411732-2413256 | YP_159076 | YP_157048 | ND | YP_159701 | YP_158658 | YP_158658 | ND | ND | ND | ND | ND |
| *Aurantimonas manganoxydans* SI85-9A1 | gb|AAPJ01000010|:22546-24020 | ZP_01227075 | ND | ND | ZP_01228208 | ZP_01226649 | ZP_01226649 | ZP_01226649 | ZP_01227595 | ZP_01227594 | ZP_01227593 | ND |
| *Azoarcus* sp. BH72 | gi|119896292:3515378-3516911 | YP_931893 | YP_933062 | ND | YP_932528 | YP_932074 | YP_932074 | YP_931555 | ND | ND | ND | ND |
| *Azotobacter vinelandii* DJ | gi|226942170:2841195-2842729 | YP_002799526 | YP_002801497 | ND | YP_002799531 | YP_002800476 | YP_002800476 | YP_002800595 | ND | YP_002799719 | ND | ND |
| *Bradyrhizobium* sp. BTAi1 | gi|148251626:1273684-1275176 | YP_001239967 | YP_001239029 | ND | YP_001243367 | YP_001241260 | YP_001243423 | YP_001241260 | ND | ND | ND | YP_001237349 |
| *Burkholderia ubonensis* Bu | gb|ABBE01000728|:139-1662 | ZP_02382303 | ZP_02380638 | ND | ZP_02379463 | ZP_02376267 | ZP_02376267 | ZP_02382428 | ND | ZP_02381206 | ZP_02380596 | ZP_02377521 |
| *Burkholderia vietnamiensis* G4 | gi|134294128:2560255-2561780 | YP_001119557 | YP_001115340 | ND | YP_001119554 | YP_001116683 | YP_001116683 | YP_001116683 | ND | ND | ND | YP_001117355 |
| *Chromobacterium violaceum* ATCC 12472 | gi|34495455:4248454-4249927 | NP_902459 | NP_901683 | ND | NP_901037 | ND | ND | NP_899842 | ND | NP_902474 | ND | ND |
| *Chromohalobacter salexigens* DSM 3043 | gi|92112136:2078081-2079554 | YP_574509 | YP_573609 | YP_574510 | YP_572964 | ND | ND | ND | YP_573927 | YP_573928 | YP_573929 | YP_572603 |
| *Citreicella* sp. SE45 | gb|ACNW01000104|:72954-74311 | ZP_05782402 | ND | ND | ZP_05779265 | ZP_05779427 | ZP_05780658 | ZP_05779994 | ZP_05779879 | ZP_05782286 | ZP_05781410 | ND |
| *Colwellia psychrerythraea* 34H | gi|71277742:4078538-4080049 | YP_270743 | ND | ND | ND | ND | ND | ND | ND | ND | ND | ND |
| *Gordonia neofelifaecis* NRRL B-59395 | FJ938167 | ZP_08206879 | ZP_08206677 | ND | ND | ZP_08205601 | ZP_08205601 | ND | ZP_08204536 | ZP_08204535 | ZP_08204534 | ZP_08205970 |
| *Hahella chejuensis* KCTC 2396 | gi|83642913:6037842-6039376 | YP_433658 | ND | YP_433657 | ND | ND | ND | YP_433259 | YP_432793 | YP_432792 | YP_432791 | YP_432790 |
| *Halomonas elongata* DSM 2581 | gi|307543589:393364-394896 | YP_003898463 | YP_003897276 | YP_003898464 | YP_003898572 | ND | ND | ND | YP_003897657 | YP_003897658 | YP_003897659 | YP_003899077 |
| *Halorhodospira halophila* SL1 | gi|121996810:952248-953785 | YP_001003639 | ND | YP_001003564 | YP_001003772 | ND | ND | ND | YP_001003298 | YP_001003299 | YP_001003300 | ND |
| *Hyphomonas neptunium* ATCC 15444 | gi|114797051:2818559-2819913 | YP_761008 | ND | ND | YP_762076 | ND | ND | ND | YP_760347 | YP_760348 | YP_760349 | YP_760350 |
| *Marinobacter algicola* DG893 | gb|ABCP01000031|:35276-36807 | ND | ND | ND | ND | ZP_01893073 | ZP_01893073 | ND | ZP_01895294 | ZP_01895293 | ZP_01895916 | ZP_01893784 |
| *Marinobacter aquaeolei* VT8 | gi|120552944:798762-800294 | YP_959074 | ND | YP_959073 | ND | ND | ND | ND | YP_957441 | YP_957442 | YP_957904 | YP_961147 |
| *Mesorhizobium loti* MAFF303099 | gi|57165207:2757518-2758978 | NP_102632 | ND | ND | NP_104862 | NP_103790 | NP_103790 | ND | ND | NP_106518 | ND | ND |
| *Methylobacterium extorquens* DM4 | gi|254558653:530014-531491 | YP_003069357 | YP_003071384 | ND | YP_003069741 | YP_003070233 | YP_003070233 | YP_003067698 | ND | ND | ND | ND |
| *Methylocella silvestris* BL2 | gi|217976200:2446870-2448344 | YP_002363556 | YP_002362992 | ND | YP_002363268 | YP_002363627 | YP_002363627 | YP_002361542 | ND | ND | ND | ND |
| *Methylosinus trichosporium* OB3b | gb|ADVE01000118|:228-1703 | ZP_06887287 | ND | ND | ZP_06889985 | ZP_06889845 | ZP_06889845 | ZP_06889845 | ND | ND | ND | ND |
| *Mycobacterium smegmatis* str. MC2 155 | gi|118467340:5027948-5029475 | ND | ND | ND | ND | YP_887767 | ND | ND | YP_888191 | YP_888190 | YP_888189 | YP_888188 |
| *Nocardiopsis dassonvillei* subsp. *dassonvillei* DSM 43111 | gi|297558985:3633025-3634544 | ND | ND | ND | ND | YP_003678006 | YP_003678006 | YP_003678006 | YP_003683098 | YP_003683099. | YP_003683100 | YP_003678094 |
| *Oceanospirillum* sp. MED92 | AY136116 | ND | ND | ND | ZP_01166180 | ND | ND | ND | ZP_01167717 | ZP_01167716 | ZP_01167715 | ZP_01167714 |
| *Phenylobacterium zucineum* HLK1 | gi|197103466:1272412-1273848 | YP_002130486 | ND | ND | YP_002129273 | ND | ND | ND | YP_002130178 | YP_002130179 | YP_002130180 | YP_002130181 |
| *Pseudonocardia* sp. P1 | ADUJ01000862 | ZP_08120323 | ZP_08120324 | ND | ND | ZP_08120658 | ZP_08120658 | ND | ZP_08123486 | ZP_08123485 | ZP_08123484 | ZP_08123483 |
| *Ralstonia eutropha* H16 | gi|116693960:867626-869160 | YP_725940 | YP_725187 | ND | YP_725943 | YP_841913 | YP_841913 | YP_841585 | ND | ND | ND | ND |
| *Ralstonia eutropha* JMP134 | gi|73537298:180337-181864 | YP_295561 | YP_294853 | ND | YP_295564 | YP_297828 | YP_299305 | YP_295857 | ND | ND | ND | YP_299757 |
| *Rhodospirillum centenum* SW | gi|289546492:3833487-3834910 | YP_002297526 | ND | ND | YP_002296793 | YP_002296547 | YP_002296547 | YP_002296547 | ND | ND | ND | ND |
| *Sinorhizobium fredii* NGR234 | gi|227820587:3621038-3622458 | YP_002825932 | YP_002823566 | ND | YP_002827757 | YP_002822554 | YP_002822554 | YP_002822554 | ND | NP_444034 | YP_002827818 | ND |
| *Sphingomonas* sp. SKA58 | gb|AAQG01000006|:168682-170110 | ZP_01302503 | ND | ND | ZP_01302932 | ZP_01305387 | ZP_01305387 | ND | ZP_01302628 | ZP_01302627 | ZP_01302626 | ZP_01302625 |
| *Stackebrandtia nassauensis* DSM 44728 | gi|291297538:5816074-5817581 | ND | ND | ND | ND | YP_003508895 | YP_003508895 | YP_003514055 | YP_003514534 | YP_003514535 | YP_003514536 | YP_003514537 |
| *Streptomyces flavogriseus* ATCC 33331 | gb|CP002475|:6290327-6291840 | ND | ND | ND | ND | ADW05905 | ADW05905 | ADW05905 | ADW06359 | ADW06358 | ADW07065 | ADW06356 |
| *Tsukamurella paurometabola* DSM 20162 | FJ468341 | ND | YP_003646237 | ND | ND | ND | ND | ND | YP_003646517 | YP_003646516 | YP_003646515 | YP_003648589 |
| *Variovorax paradoxus* EPS | gi|319790694:5899432-5900954 | YP_004156160 | YP_004157584 | ND | YP_004155631 | YP_004156929 | YP_004156929 | YP_004155740 | ND | ND | ND | YP_004156269 |
| *Vibrio brasiliensis* LMG 20546 | NZ_AEVS01000097 | ZP_08097013 | ND | ZP_08097014 | ND | ND | ND | ND | ZP_08097933 | ZP_08097932 | ZP_08097931 | ND |
| *Xanthobacter autotrophicus* Py2 | gi|154243958:2150387-2151864 | YP_001418409 | YP_001418139 | ND | YP_001417996 | YP_001418230 | YP_001418230 | YP_001418230 | ND | ND | ND | ND |
| *Halomonas* sp. TD01 | JF340230 | EGP20415 | EGP19504 | EGP20414 | EGP21321 | EGP20509 | EGP18355 | EGP19590 | EGP18461 | EGP18460 | EGP18459 | EGP18127 |

ND, not determined. Accession numbers are retrieved from GenBank.
