# Supplementary figures and images for: Comparative genomics study of polyhydroxyalkanoates (PHA) and ectoine relevant genes from Halomonas sp. TD01 revealed extensive horizontal gene transfer events and co-evolutionary relationships
Source: Microb Cell Fact. 2011 Nov 1;10:88. doi: 10.1186/1475-2859-10-88 (PMC3227634; doi:10.1186/1475-2859-10-88)

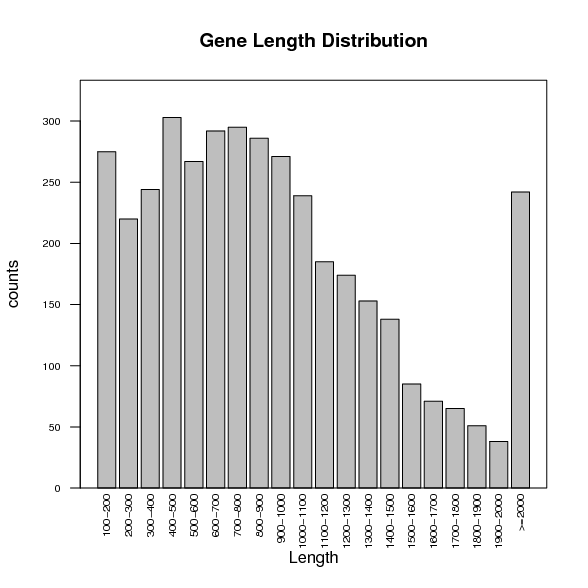


## Figure S1 - Gene length distribution of *Halomonas* sp. TD1.

Supplement: Additional file 2 — Figure S1. Gene length distribution of Halomonas sp. TD1. [file 1475-2859-10-88-S2.DOC]
