# Supplementary material for: Comparative genomics study of polyhydroxyalkanoates (PHA) and ectoine relevant genes from Halomonas sp. TD01 revealed extensive horizontal gene transfer events and co-evolutionary relationships
Source: Microb Cell Fact. 2011 Nov 1;10:88. doi: 10.1186/1475-2859-10-88 (PMC3227634; doi:10.1186/1475-2859-10-88)
Supplement: Additional file 3 — Figure S2. COG function and KEGG pathway classification of Halomonas sp. TD1. [file 1475-2859-10-88-S3.DOC]

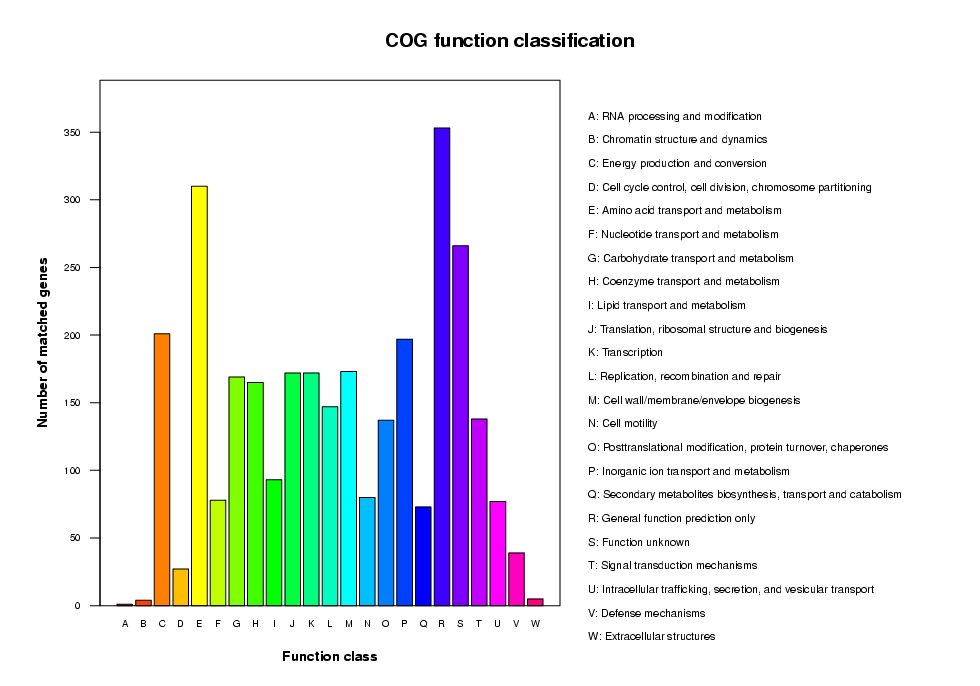


## Figure S2A - COG function classification of *Halomonas* sp. TD1.

The COG function of proteins from *Halomonas* sp. TD1 was classified according to the BLAST results.


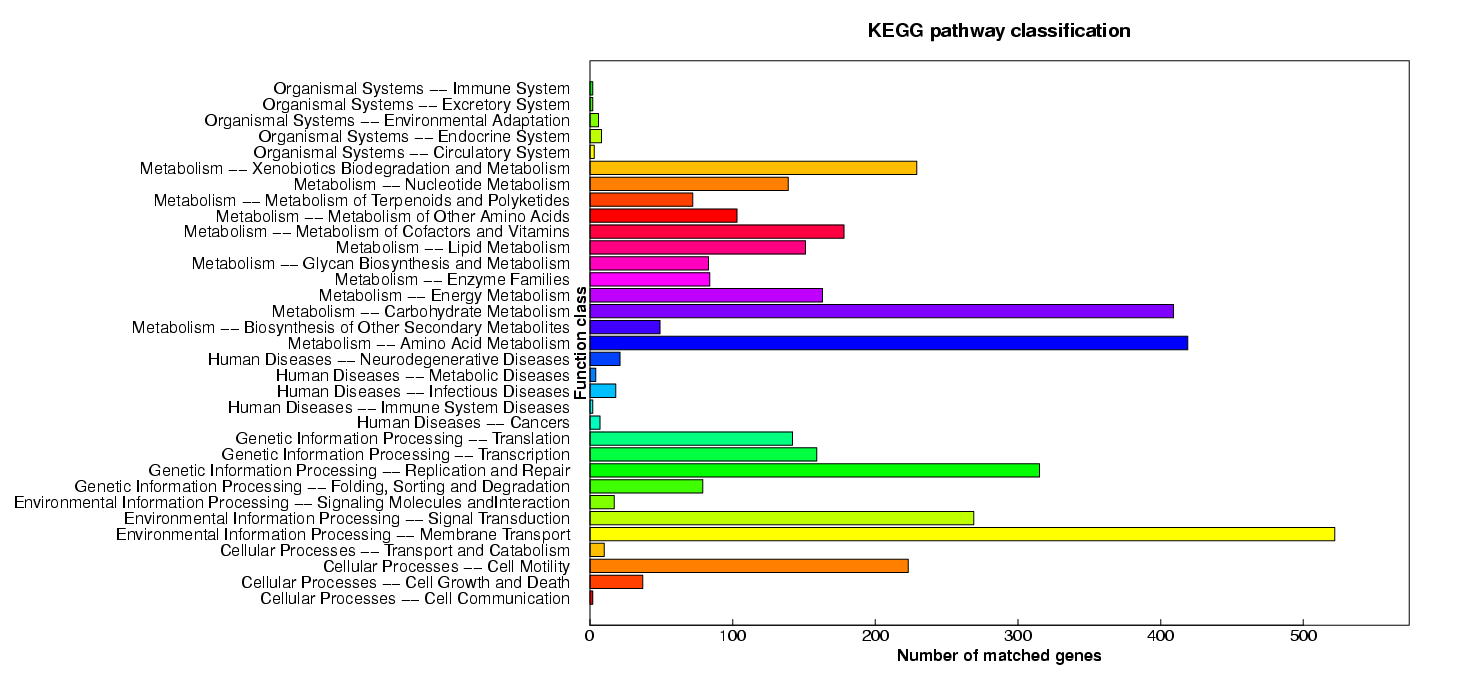


## Figure S2B - KEGG pathway classification of *Halomonas* sp. TD1.

The pathway classification of *Halomonas* sp. TD01 according to the principle of KEGG (Kyoto Encyclopedia of Genes and Genomes, www.genome.jp/kegg/)
