# Supplementary material for: Comparative genomics study of polyhydroxyalkanoates (PHA) and ectoine relevant genes from Halomonas sp. TD01 revealed extensive horizontal gene transfer events and co-evolutionary relationships
Source: Microb Cell Fact. 2011 Nov 1;10:88. doi: 10.1186/1475-2859-10-88 (PMC3227634; doi:10.1186/1475-2859-10-88)
Supplement: Additional file 4 — Figure S3. Alignment of the chromosomes of C. salexigens and Halomonas sp. TD1. [file 1475-2859-10-88-S4.DOC]

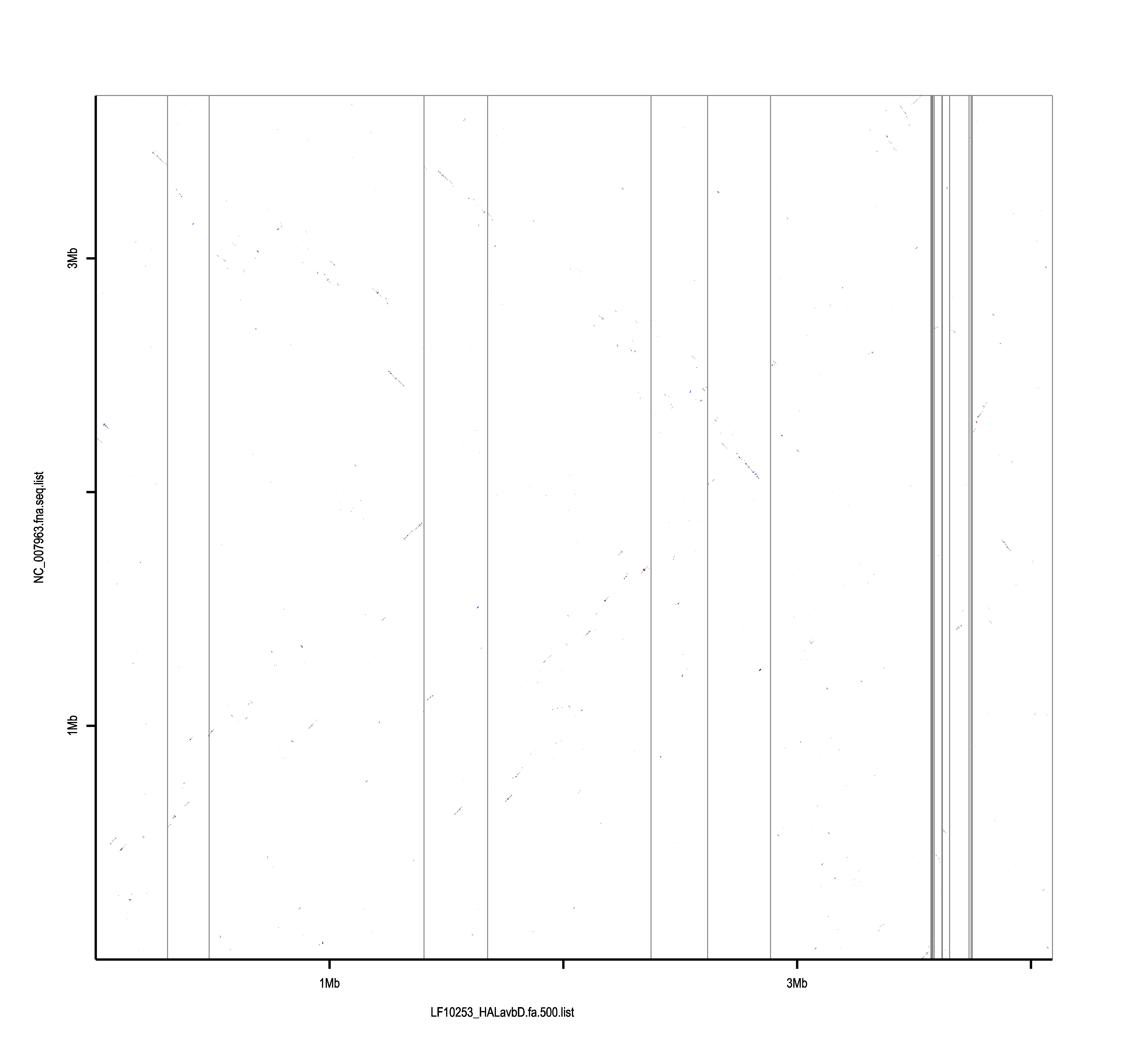


## Figure S3 - Alignment of the chromosomes of *C. salexigens* and *Halomonas* sp. TD1.

The proteins of each strain were aligned with BLAST, and the best hits were selected. The Y axis was protein set of *C. salexigens* according to the sequence of chromosome, and the X axis was protein set of *Halomonas* sp. TD1 according to sequence of the scaffolds.
