# Supplementary material for: Comparative genomics study of polyhydroxyalkanoates (PHA) and ectoine relevant genes from Halomonas sp. TD01 revealed extensive horizontal gene transfer events and co-evolutionary relationships
Source: Microb Cell Fact. 2011 Nov 1;10:88. doi: 10.1186/1475-2859-10-88 (PMC3227634; doi:10.1186/1475-2859-10-88)
Supplement: Additional file 5 — Table S2. Evolutionary distances of 16S rDNA, PHA and ectoine relevant proteins between Halomonas sp. TD01 and other species. [file 1475-2859-10-88-S5.DOC]

**Table S2. Evolutionary distances of 16S rDNA, PHA and ectoine relevant proteins between *Halomonas* sp. TD01 and other species**

| Plylum | Class | Order | Family | Species | 16S rDNA | PhaC1 | PhaC2 | PhaP | PhaR | PhaZ1 | PhaZ2 | PhaZ3 | EctA | EctB | EctC | EctD |
| --- | --- | --- | --- | --- | --- | --- | --- | --- | --- | --- | --- | --- | --- | --- | --- | --- |
| *Actinobacteria* | *Actinobacteria* | *Actinomycetales* | *Corynebacterineae* | *Gordonia neofelifaecis* NRRL B-59395 | 0.257 | 0.982 | 1.451 | ND | ND | 1.295 | 1.345 | ND | 1.001 | 0.612 | 0.746 | 0.724 |
|  |  |  | *Pseudonocardiaceae* | *Pseudonocardia* sp. P1 | 0.272 | 0.897 | 1.520 | ND | ND | 0.996 | 1.326 | ND | 0.824 | 0.634 | 0.765 | 0.625 |
|  |  |  | *Tsukamurellaceae* | *Tsukamurella paurometabola* DSM 20162 | 0.246 | ND | 1.856 | ND | ND | ND | ND | ND | 0.801 | 0.623 | 0.746 | 0.734 |
|  |  |  | *Glycomycetaceae* | *Stackebrandtia nassauensis* DSM 44728 | 1.381 | ND | ND | ND | ND | 1.501 | 1.366 | 1.161 | 0.824 | 0.623 | 0.728 | 0.664 |
|  |  |  | *Mycobacteriaceae* | *Mycobacterium smegmatis* str. MC2 155 | 1.357 | ND | ND | ND | ND | 1.206 | ND | ND | 0.896 | 0.669 | 0.822 | 0.683 |
|  |  |  | *Nocardiopsaceae* | *Nocardiopsis dassonvillei* subsp. *dassonvillei* DSM 43111 | 1.357 | ND | ND | ND | ND | 1.013 | 0.863 | 2.321 | 0.735 | 0.590 | 0.746 | 0.673 |
|  |  |  | *Streptomycetaceae* | *Streptomyces flavogriseus* ATCC 33331 | 0.249 | ND | ND | ND | ND | 1.124 | 0.863 | 2.230 | 0.801 | 0.629 | 0.765 | 0.607 |
| *Proteobacteria* | *Alphaproteobacteria* | *Caulobacterales* | *Caulobacteraceae* | *Phenylobacterium zucineum* HLK1 | 0.207 | 0.785 | ND | ND | 1.099 | ND | ND | ND | 0.714 | 0.595 | 0.549 | 0.664 |
|  |  | *Rhizobiales* | *Aurantimonadaceae* | *Aurantimonas manganoxydans* SI85-9A1 | 0.202 | 0.767 | ND | ND | 1.159 | 0.502 | 1.099 | 2.188 | 0.871 | 0.652 | 0.549 | ND |
|  |  |  | *Beijerinckiaceae* | *Methylocella silvestris* BL2 | 1.233 | 0.762 | 0.506 | ND | 1.070 | 0.461 | 1.130 | 2.321 | ND | ND | ND | ND |
|  |  |  | *Bradyrhizobiaceae* | *Bradyrhizobium* sp. BTAi1 | 0.225 | 0.736 | 0.445 | ND | 1.014 | 0.412 | 0.623 | 2.188 | ND | ND | ND | 1.094 |
|  |  |  | *Methylobacteriaceae* | *Methylobacterium extorquens* DM4 | 1.247 | 0.719 | 0.687 | ND | 1.128 | 0.461 | 1.068 | 2.070 | ND | ND | ND | ND |
|  |  |  | *Methylocystaceae* | *Methylosinus trichosporium* OB3b | 0.213 | 0.767 | ND | ND | 1.070 | 0.662 | 1.232 | 2.188 | ND | ND | ND | ND |
|  |  |  | *Phyllobacteriaceae* | *Mesorhizobium loti* MAFF303099 | 1.286 | 0.818 | ND | ND | 1.191 | 1.067 | 0.478 | ND | ND | 0.762 | ND | ND |
|  |  |  | *Rhizobiaceae* | *Sinorhizobium fredii* NGR234 | 0.216 | 0.745 | 0.402 | ND | 1.041 | 1.185 | 0.521 | 2.188 | ND | 0.595 | 0.903 | ND |
|  |  |  | *Xanthobacteraceae* | *Xanthobacter autotrophicus* Py2 | 0.202 | 0.689 | 0.609 | ND | 1.099 | 0.534 | 1.147 | 2.188 | ND | ND | ND | ND |
|  |  | *Rhodobacterales* | *Hyphomonadaceae* | *Hyphomonas neptunium* ATCC 15444 | 1.251 | 0.897 | ND | ND | 1.128 | ND | ND | ND | 0.735 | 0.584 | 0.595 | 0.644 |
|  |  |  | *Rhodobacteraceae* | *Citreicella* sp. SE45 | 0.232 | 0.842 | ND | ND | 1.293 | 0.579 | 0.940 | 2.230 | 0.824 | 0.601 | 0.676 | ND |
|  |  | *Rhodospirillales* | *Acetobacteraceae* | *Acidiphilium cryptum* JF-5 | 1.268 | 0.664 | 0.627 | ND | 1.191 | 1.144 | 0.815 | ND | 0.871 | 0.590 | 0.711 | 0.607 |
|  |  |  | *Rhodospirillaceae* | *Rhodospirillum centenum* SW | 1.222 | 0.624 | ND | ND | 1.191 | 0.502 | 1.197 | 2.188 | ND | ND | ND | ND |
|  |  | *Sphingomonadales* | *Sphingomonadaceae* | *Sphingomonas* sp. SKA58 | 0.221 | 0.785 | ND | ND | 1.224 | 0.739 | 1.180 | ND | 0.615 | 0.552 | 0.746 | 0.644 |
|  | *Betaproteobacteria* | *Burkholderiales* | *Alcaligenaceae* | *Achromobacter piechaudii* ATCC 43553 | 1.283 | 0.886 | 0.343 | ND | 0.987 | 0.451 | 1.130 | 2.034 | 0.824 | 0.573 | 0.626 | 0.598 |
|  |  |  |  | *Achromobacter xylosoxidans* A8 | 0.177 | 0.891 | 0.330 | ND | 1.014 | 0.945 | 1.473 | 2.070 | 0.778 | 0.568 | 0.643 | 0.607 |
|  |  |  | *Burkholderiaceae* | *Burkholderia vietnamiensis* G4 | 1.251 | 0.856 | 0.393 | ND | 0.987 | 0.794 | 1.250 | 2.422 | ND | ND | ND | 1.470 |
|  |  |  |  | *Burkholderia ubonensis* Bu | 1.249 | 0.813 | 1.579 | ND | 1.014 | 0.794 | 1.287 | 1.176 | ND | 1.003 | 0.862 | 1.366 |
|  |  |  |  | *Ralstonia eutropha* JMP134 | 0.192 | 0.808 | 0.511 | ND | 1.070 | 0.882 | 1.197 | 1.359 | ND | ND | ND | 1.427 |
|  |  |  |  | *Ralstonia eutropha* H16 | 0.188 | 0.794 | 0.527 | ND | 1.070 | 0.913 | 1.269 | 1.999 | ND | ND | ND | ND |
|  |  |  | *Comamonadaceae* | *Variovorax paradoxus* EPS | 1.283 | 0.876 | 1.761 | ND | 1.070 | 0.780 | 1.269 | 1.272 | ND | ND | ND | 1.050 |
|  |  | *Neisseriales* | *Neisseriaceae* | *Chromobacterium violaceum* ATCC 12472 | 1.280 | 0.837 | 0.527 | ND | 1.128 | ND | ND | 1.022 | ND | 0.850 | ND | ND |
|  |  | *Rhodocyclales* | *Rhodocyclaceae* | *Azoarcus* sp. BH72 | 1.267 | 0.632 | 0.511 | ND | 0.961 | 0.962 | 1.180 | 1.146 | ND | ND | ND | ND |
|  |  |  |  | *Aromatoleum aromaticum* EbN1 | 0.172 | 0.632 | 0.174 | ND | 0.987 | 0.412 | 1.180 | ND | ND | ND | ND | ND |
|  | *Gammaproteobacteria* | *Alteromonadales* | *Alteromonadaceae* | *Marinobacter algicola* DG893 | 1.267 | ND | ND | ND | ND | 1.393 | 1.269 | ND | 0.634 | 0.563 | 0.842 | 0.607 |
|  |  |  |  | *Marinobacter aquaeolei* VT8 | 0.097 | 0.758 | ND | 1.600 | ND | ND | ND | ND | 0.615 | 0.573 | 0.783 | 0.562 |
|  |  |  | *Colwelliaceae* | *Colwellia psychrerythraea* 34H | 1.323 | 0.652 | ND | ND | ND | ND | ND | ND | ND | ND | ND | ND |
|  |  | *Chromatiales* | *Chromatiaceae* | *Allochromatium vinosum* DSM 180 | 1.260 | ND | 1.798 | ND | 0.936 | 0.808 | 1.306 | 2.108 | 1.218 | ND | ND | ND |
|  |  |  | *Ectothiorhodospiraceae* | *Alkalilimnicola ehrlichii* MLHE-1 | 0.118 | ND | 1.641 | 1.061 | 0.841 | ND | ND | ND | 0.801 | 0.711 | 0.765 | 0.810 |
|  |  |  |  | *Halorhodospira halophila* SL1 | 1.285 | 0.672 | ND | 1.210 | 0.775 | ND | ND | ND | 0.921 | 0.612 | 0.802 | ND |
|  |  | *Oceanospirillales* | *Hahellaceae* | *Hahella chejuensis* KCTC 2396 | 1.331 | 0.546 | ND | 1.118 | ND | ND | ND | 1.176 | 0.778 | 0.563 | 0.534 | 0.553 |
|  |  |  | *Halomonadaceae* | *Chromohalobacter salexigens* DSM 3043 | 0.046 | 0.312 | 0.211 | 0.455 | 0.194 | ND | ND | ND | 0.108 | 0.179 | 0.158 | 0.308 |
|  |  |  |  | *Halomonas elongata* DSM 2581 | 0.040 | 0.191 | 0.185 | 0.382 | 0.159 | ND | ND | ND | 0.063 | 0.119 | 0.200 | 0.294 |
|  |  |  | *Oceanospirillaceae* | *Oceanospirillum* sp. MED92 | 0.113 | ND | ND | ND | 0.961 | ND | ND | ND | 0.974 | 0.516 | 0.477 | 0.664 |
|  |  | *Pseudomonadales* | *Pseudomonadaceae* | *Azotobacter vinelandii* DJ | 1.308 | 0.847 | 0.570 | ND | 0.864 | 0.412 | 1.163 | 1.132 | ND | 0.924 | ND | ND |
|  |  | *Vibrionales* | *Vibrionaceae* | *Vibrio brasiliensis* LMG 20546 | 0.134 | 0.685 | ND | 1.033 | ND | ND | ND | ND | 0.847 | 0.584 | 0.505 | ND |

The trees were constructed using the neighbor-joining algorithm with MEGA (version 5.03) software with bootstrap values based on 500 replications. ND, not determined.
