# Supplementary material for: Comparative genomics study of polyhydroxyalkanoates (PHA) and ectoine relevant genes from Halomonas sp. TD01 revealed extensive horizontal gene transfer events and co-evolutionary relationships
Source: Microb Cell Fact. 2011 Nov 1;10:88. doi: 10.1186/1475-2859-10-88 (PMC3227634; doi:10.1186/1475-2859-10-88)
Supplement: Additional file 6 — Figure S4. Signal peptide prediction of PhaZ3 with neural networks (NN) and hidden markov models (HMM). [file 1475-2859-10-88-S6.DOC]

|  |  | SignalP 3.0 Server - prediction resultsTechnical University of Denmark |
| --- | --- | --- |

Using neural networks (NN) and hidden Markov models (HMM) trained on Gram-positive bacteria

**>Sequence**

**SignalP-NN result:**


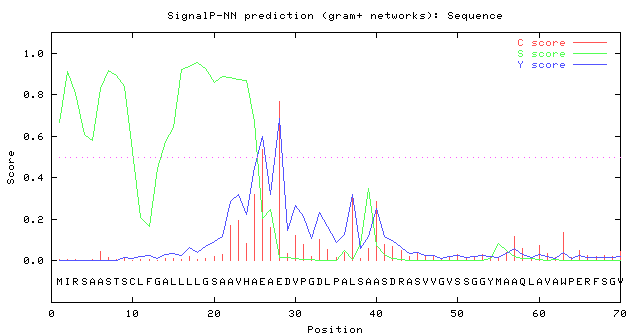


**Figure S4A - Signal peptide prediction of PhaZ3 with neural networks (NN)**

# [data](http://www.cbs.dtu.dk/services/SignalP/tmp/TMP110904092114.1325108/nn-score.1)

>Sequence length = 70

# Measure Position Value Cutoff signal peptide?

max. C 28 0.766 0.52 YES

max. Y 28 0.688 0.32 YES

max. S 18 0.954 0.97 NO

mean S 1-27 0.698 0.51 YES

D 1-27 0.693 0.45 YES

# Most likely cleavage site between pos. 27 and 28: AEA-ED

**SignalP-HMM result:**


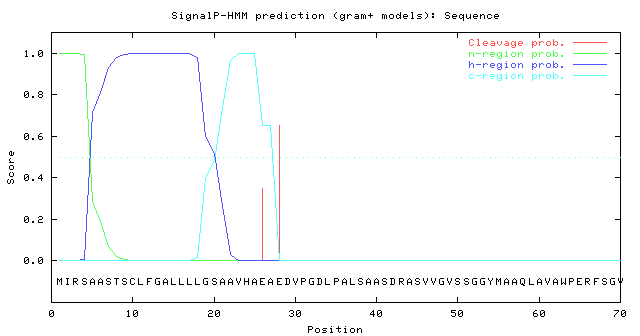


**Figure S4B - Signal peptide prediction of PhaZ3 with hidden markov models (HMM)**

# [data](http://www.cbs.dtu.dk/services/SignalP/tmp/TMP110904092114.1325108/hmm-score.1)

>Sequence

Prediction: Signal peptide

Signal peptide probability: 1.000

Max cleavage site probability: 0.650 between pos. 27 and 28

# [gnuplot script](http://www.cbs.dtu.dk/services/SignalP/tmp/TMP110904092114.1325108/plot.gnu)

for making the plot(s)

[**Explain**](http://www.cbs.dtu.dk/services/SignalP-3.0/output.html) the output. Go [**back**](javascript:history.back()).
