# Supplementary material for: Comparative genomics study of polyhydroxyalkanoates (PHA) and ectoine relevant genes from Halomonas sp. TD01 revealed extensive horizontal gene transfer events and co-evolutionary relationships
Source: Microb Cell Fact. 2011 Nov 1;10:88. doi: 10.1186/1475-2859-10-88 (PMC3227634; doi:10.1186/1475-2859-10-88)
Supplement: Additional file 7 — Figure S5. Calculated isoelectric point (pI) distribution. Isoelectric point (pI) distribution of halophilic bacteria (Halomonas sp. TD01 and Halomonas elongata DSM 2581), haloarchaea (Haloarcula marismortui ATCC 43049) and non-halophilic bacterium (Escherichia coli MG1655) versus percentage of total proteins. Distribution with the interval of 0.2 pI was counted and plotted. [file 1475-2859-10-88-S7.DOC]

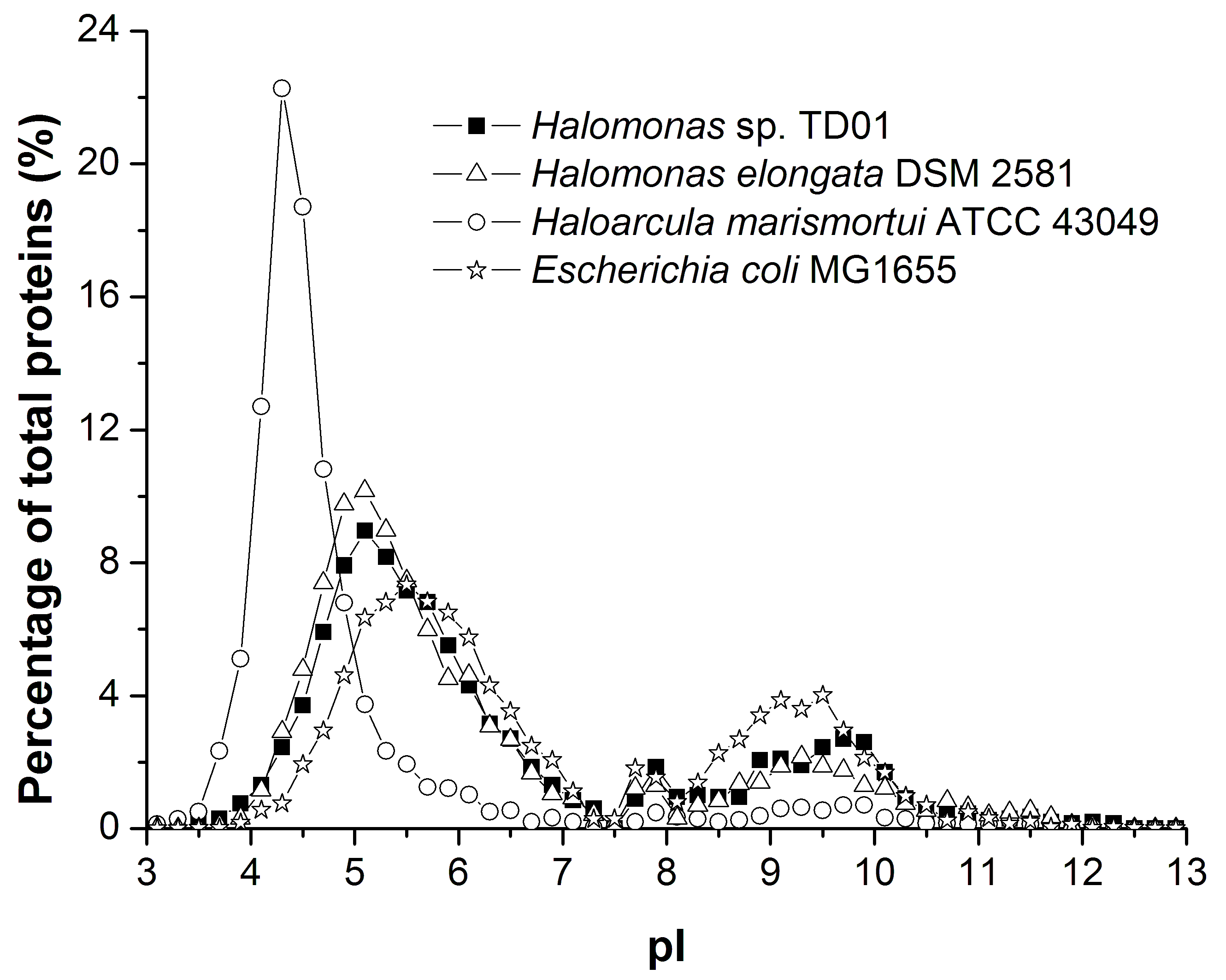


## Figure S5 - Calculated isoelectric point (pI) distribution.

Isoelectric point (pI) distribution of halophilic bacteria (*Halomonas* sp. TD01 and *Halomonas elongata* DSM 2581), haloarchaea (*Haloarcula marismortui* ATCC 43049) and non-halophilic bacterium (*Escherichia coli* MG1655) versus percentage of total proteins. Distribution with the interval of 0.2 pI was counted and plotted
