# Supplementary material for: Comparative genomics study of polyhydroxyalkanoates (PHA) and ectoine relevant genes from Halomonas sp. TD01 revealed extensive horizontal gene transfer events and co-evolutionary relationships
Source: Microb Cell Fact. 2011 Nov 1;10:88. doi: 10.1186/1475-2859-10-88 (PMC3227634; doi:10.1186/1475-2859-10-88)
Supplement: Additional file 8 — Table S3. Accession numbers of putative PhaA, PhaB and osmolytes relevant enzymes in the genome of Halomonas sp. TD01. [file 1475-2859-10-88-S8.DOC]

**Table S3. Accession numbers of putative PhaA, PhaB and osmolytes relevant enzymes in the genome of *Halomonas* sp. TD01**

|  | Enzyme | | Gene | Accession Number^a^ |
| --- | --- | --- | --- | --- |
| PHA | Acetyl-CoA acetyltransferase | | *phaA1* | EGP19848 |
|  |  | | *phaA2* | EGP17993 |
|  |  | | *phaA3* | EGP19165 |
|  |  | | *phaA4* | EGP20211 |
|  |  | | *phaA5* | EGP19200 |
|  |  | | *phaA6* | EGP17984 |
|  | 3-hydroxybutyryl-CoA dehydrogenase | | *phaB1* | EGP20949 |
|  |  | | *phaB2* | EGP20436 |
| Ectoine | Transporter | | *teaA* | EGP19595 |
|  | Ectoine hydrolase | | *doeA* | EGP21610 |
|  | N-acetyl diamonbutyric acid acylase | | *doeB* | EGP21611 |
|  | Aspartate-semialdehyde dehydrogenase | | *doeC* | EGP21617 |
|  | Diaminobutyric acid transaminase | | *doeD* | EGP21618 |
| Betaine | Synthesis | | *betABI* | EGP18213, EGP18214, EGP18215 |
|  |  | | *betB* | EGP20127 |
|  | Transporter | | *BCCT1* | EGP21593 |
|  |  | | *BCCT2* | EGP21520 |
|  |  | | *BCCT3* | EGP20946, EGP20947 |
|  |  | | *BCCT4* | EGP19787 |
|  |  | | *BCCT5* | EGP19787 |
|  |  | | *BCCT6* | EGP19791 |
|  |  | | *BCCT7* | EGP18938 |
|  |  | | *BCCT8* | EGP18088 |
|  |  | | *proU1* | EGP21521, EGP21522, EGP21523 |
|  |  | | *proU2* | EGP20944 |
|  |  | | *proU3* | EGP20282, EGP20283, EGP20282, EGP20283 |
|  |  | *proU4* | | EGP18591, EGP18592, EGP18593, EGP18594 |
|  |  | | *proU5* | EGP18216 |

^a^, GenBank accession number.
