# Supplementary material for: Comparative genomics study of polyhydroxyalkanoates (PHA) and ectoine relevant genes from Halomonas sp. TD01 revealed extensive horizontal gene transfer events and co-evolutionary relationships
Source: Microb Cell Fact. 2011 Nov 1;10:88. doi: 10.1186/1475-2859-10-88 (PMC3227634; doi:10.1186/1475-2859-10-88)
Supplement: Additional file 9 — Figure S6. Phylogenetic trees based on the PhaC1 (A), PhaC2 (B), PhaP (C), PhaR (D), PhaZ1 (E), PhaZ2 (F), PhaZ3 (G), EctA (H), EctB (I), EctC (J) and EctD (K) sequences of Halomonas sp. TD01 with their homologues of other strains. [file 1475-2859-10-88-S9.DOC]

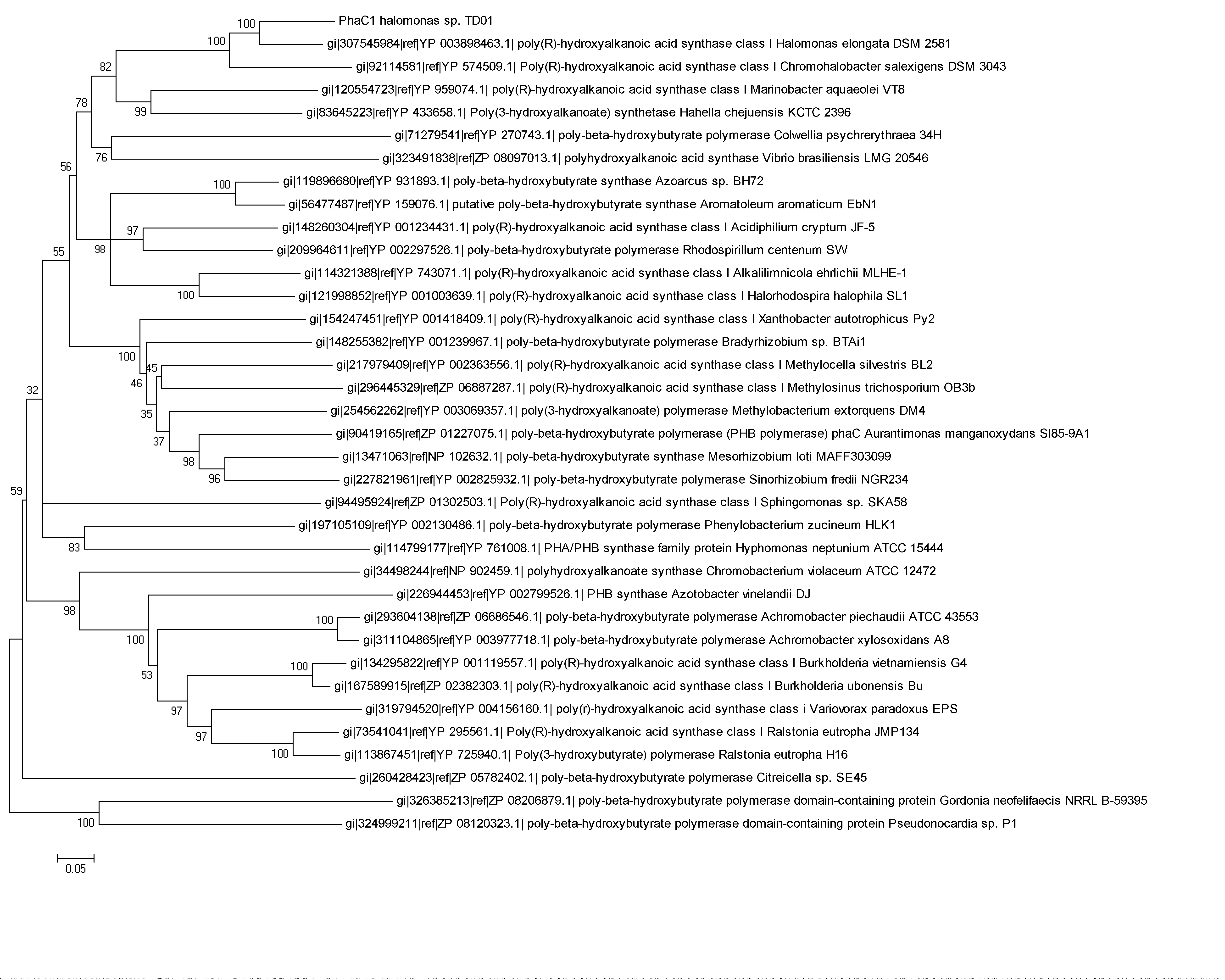


**A**

**B**


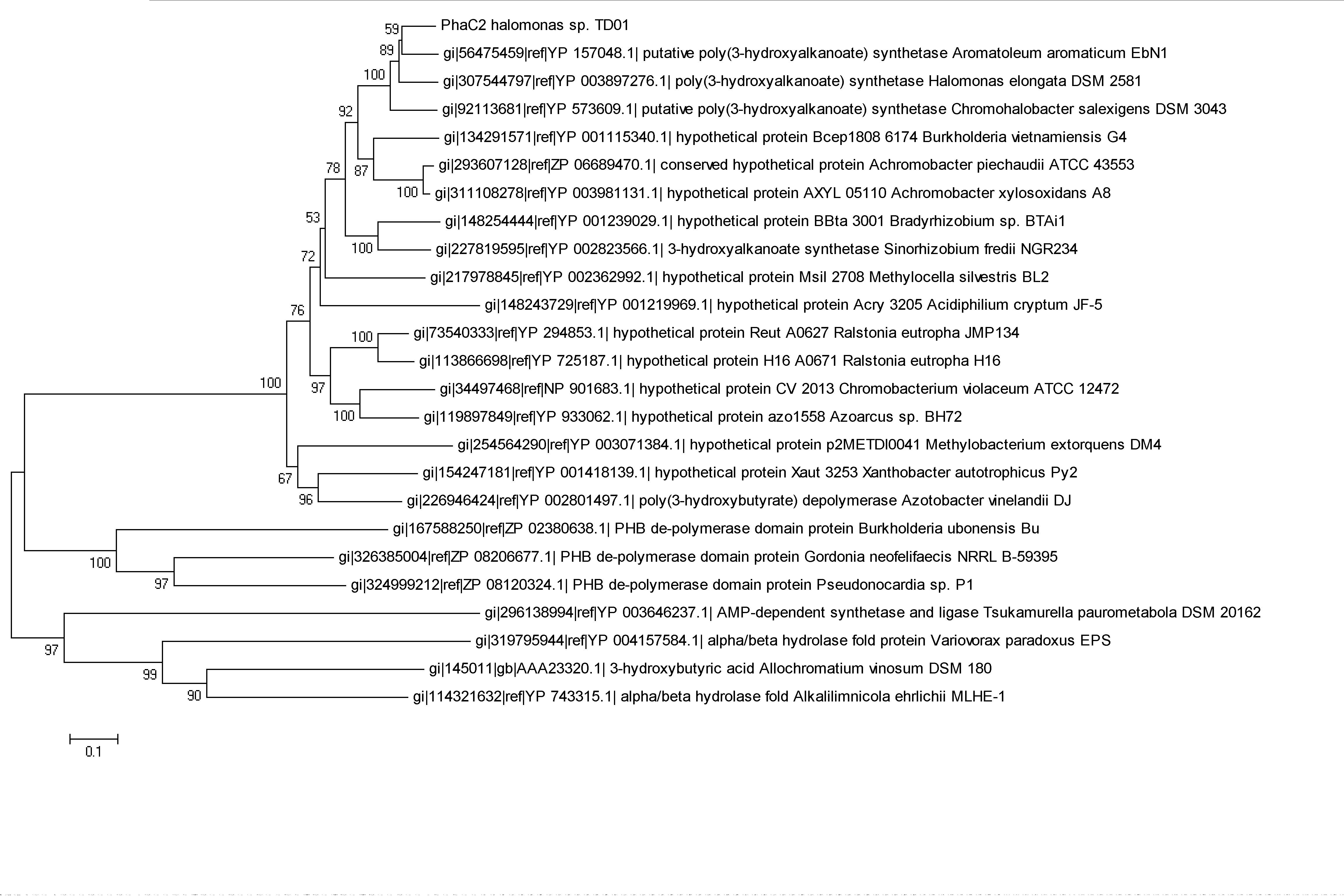


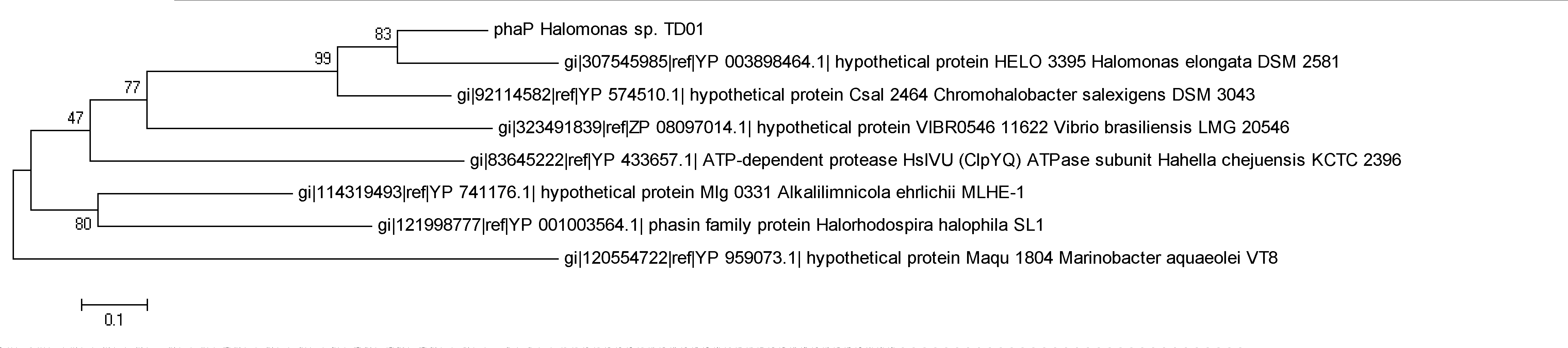


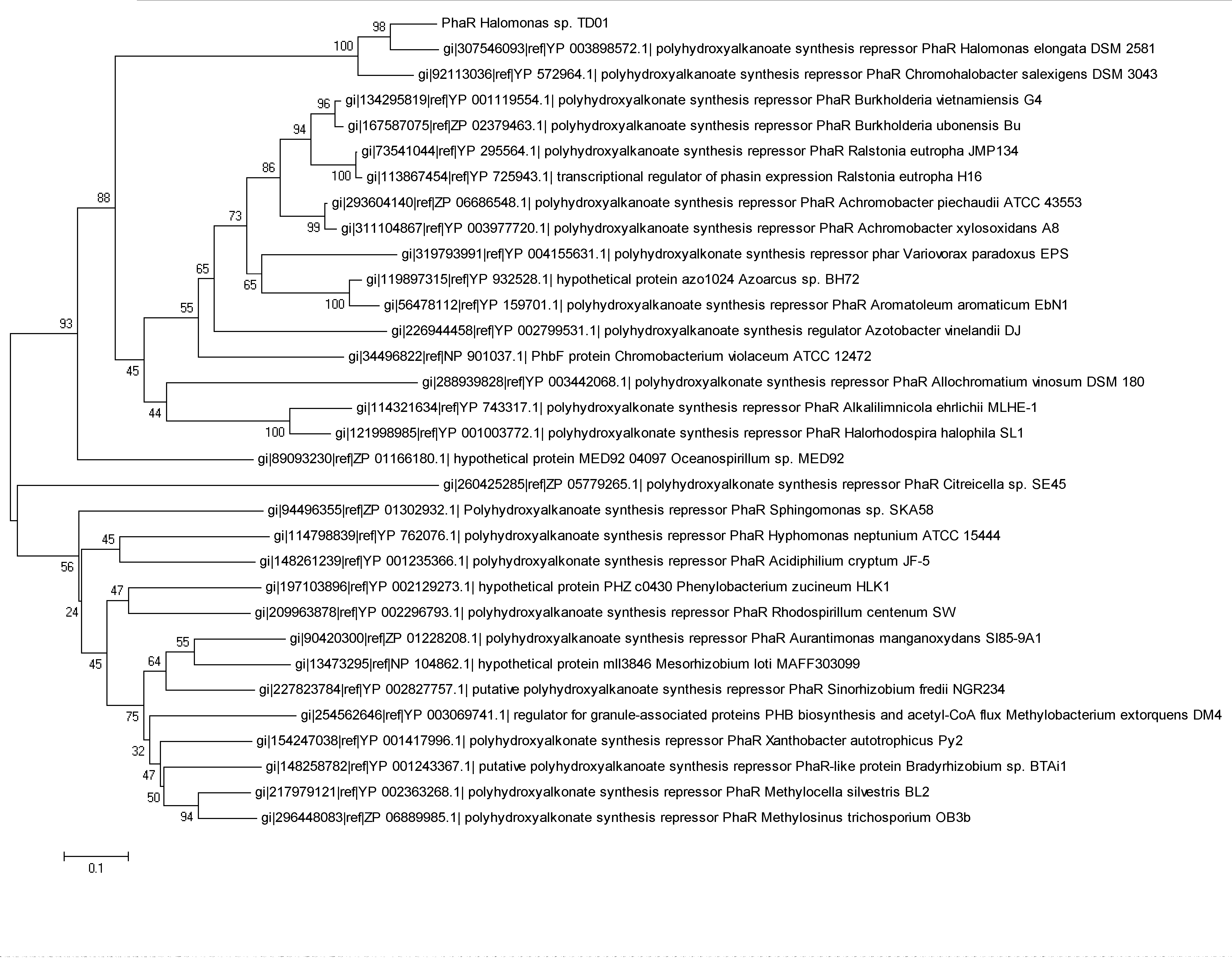


**C**

**D**


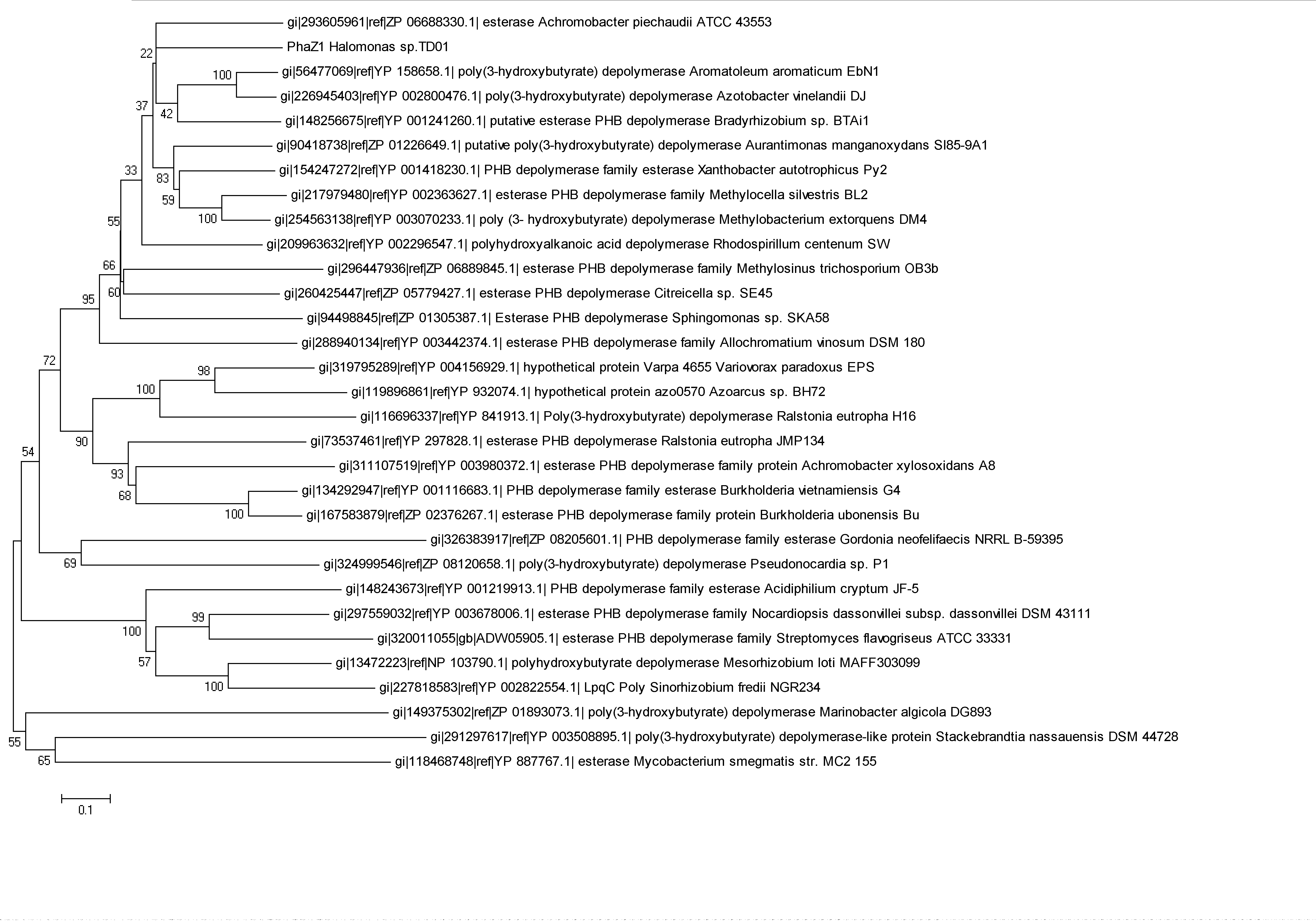


**E**


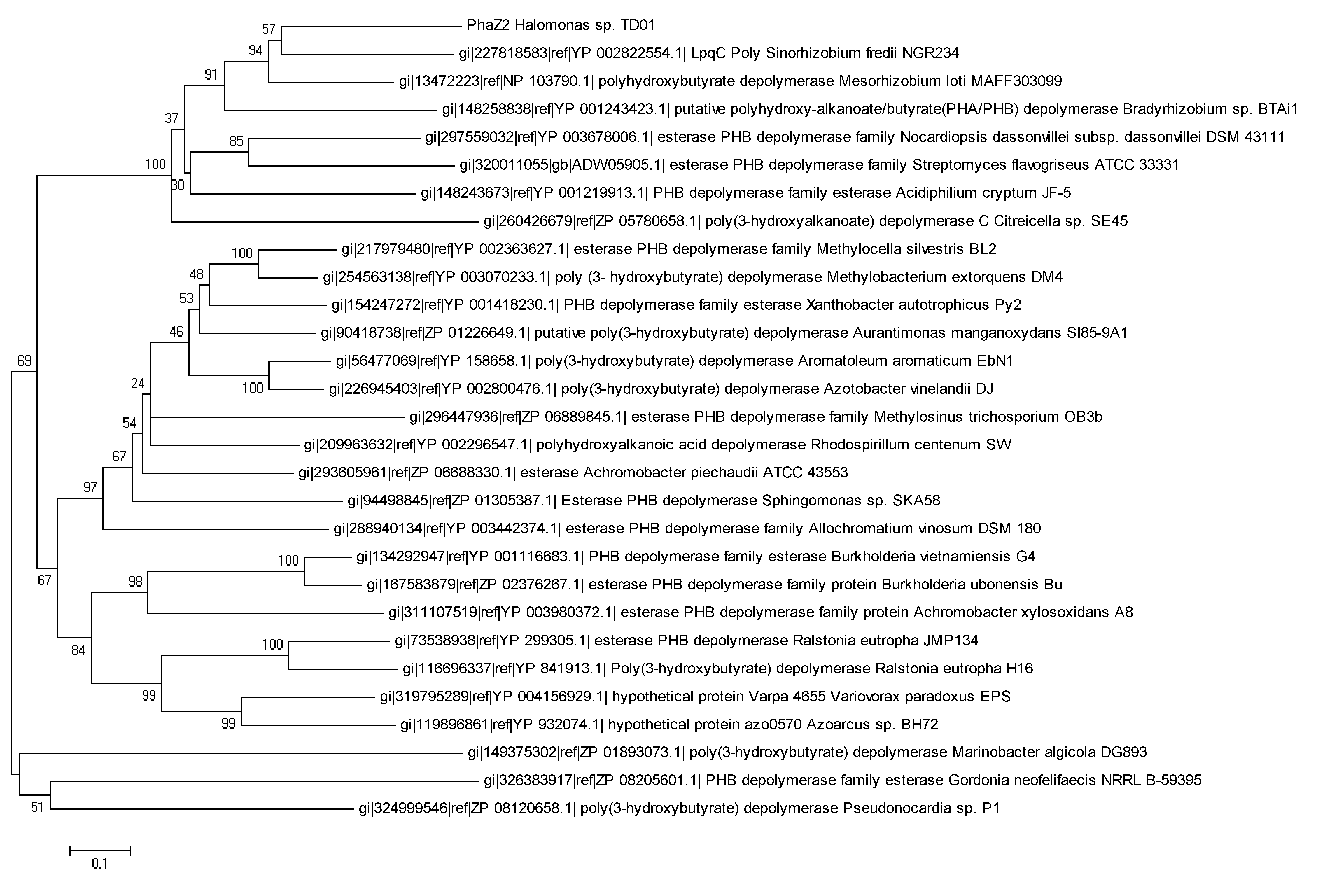


**F**


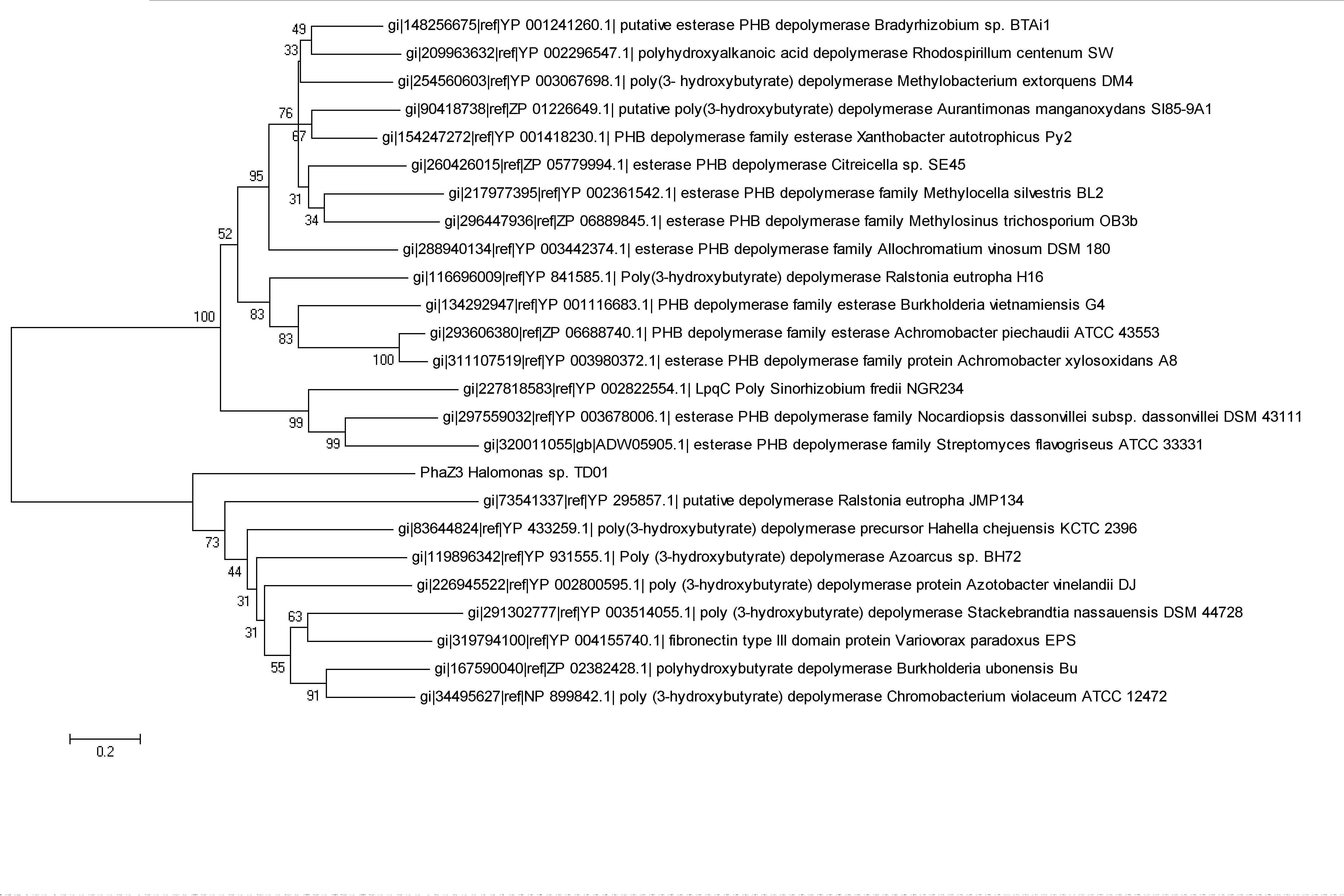


**G**


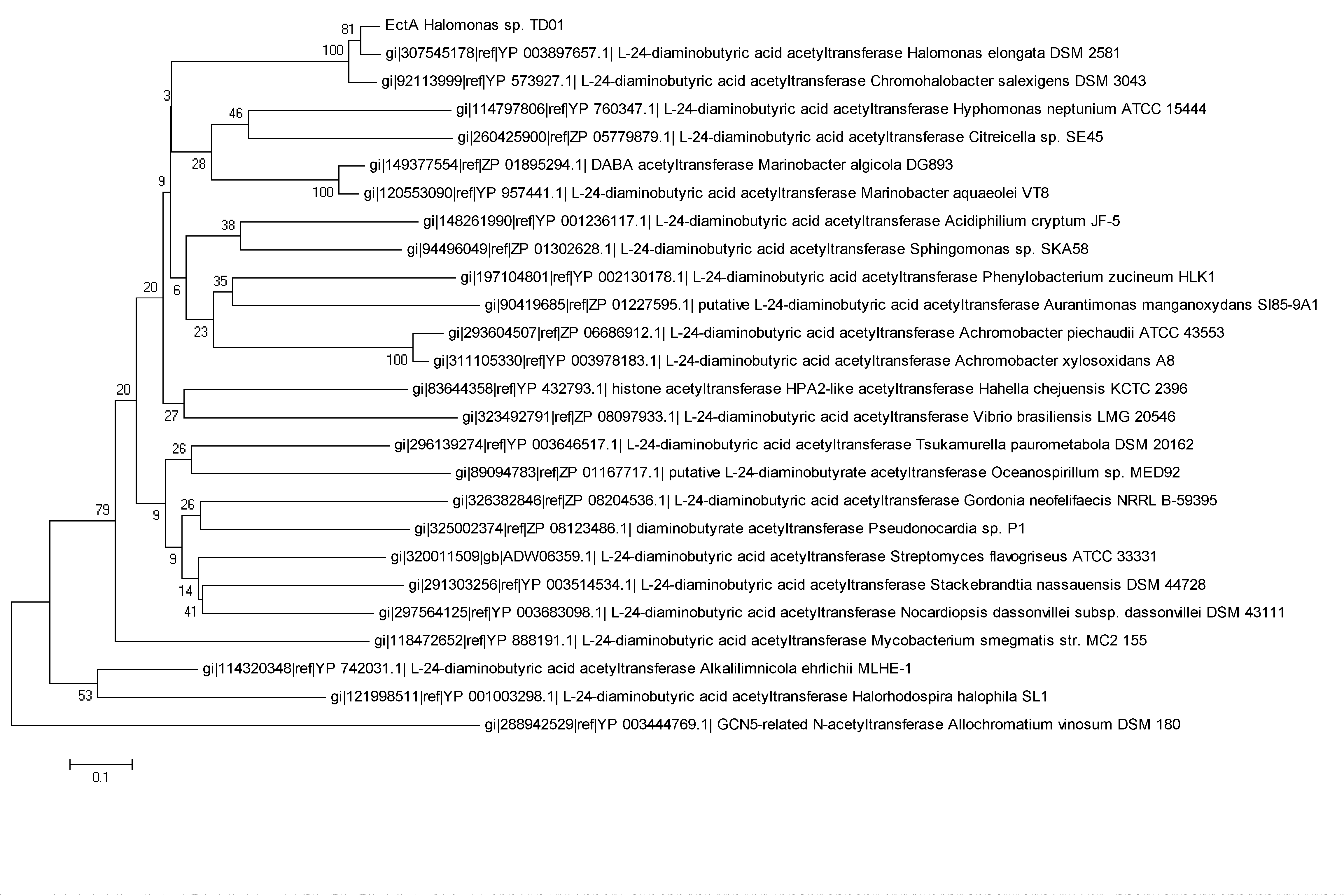


**H**


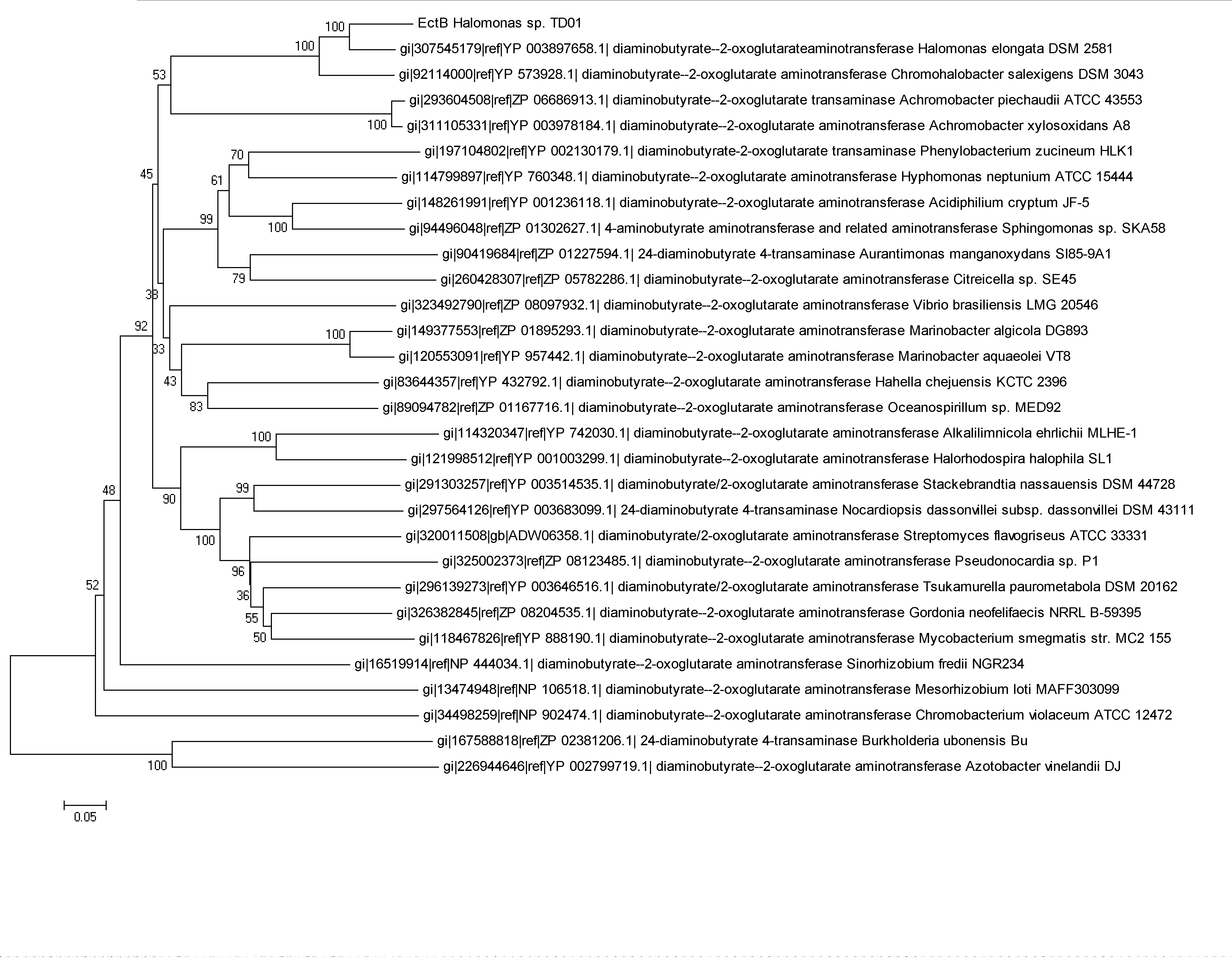


**I**


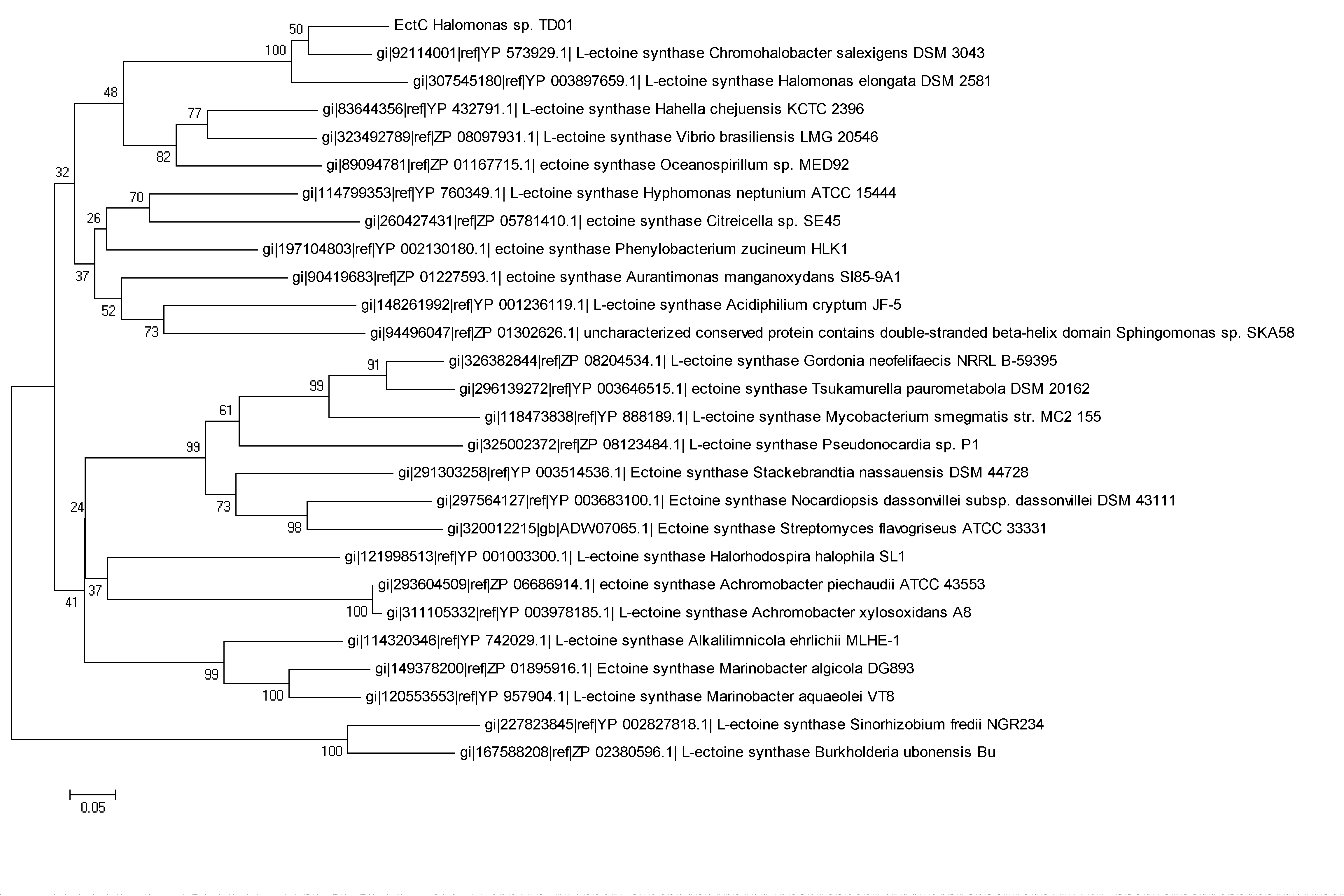


**J**


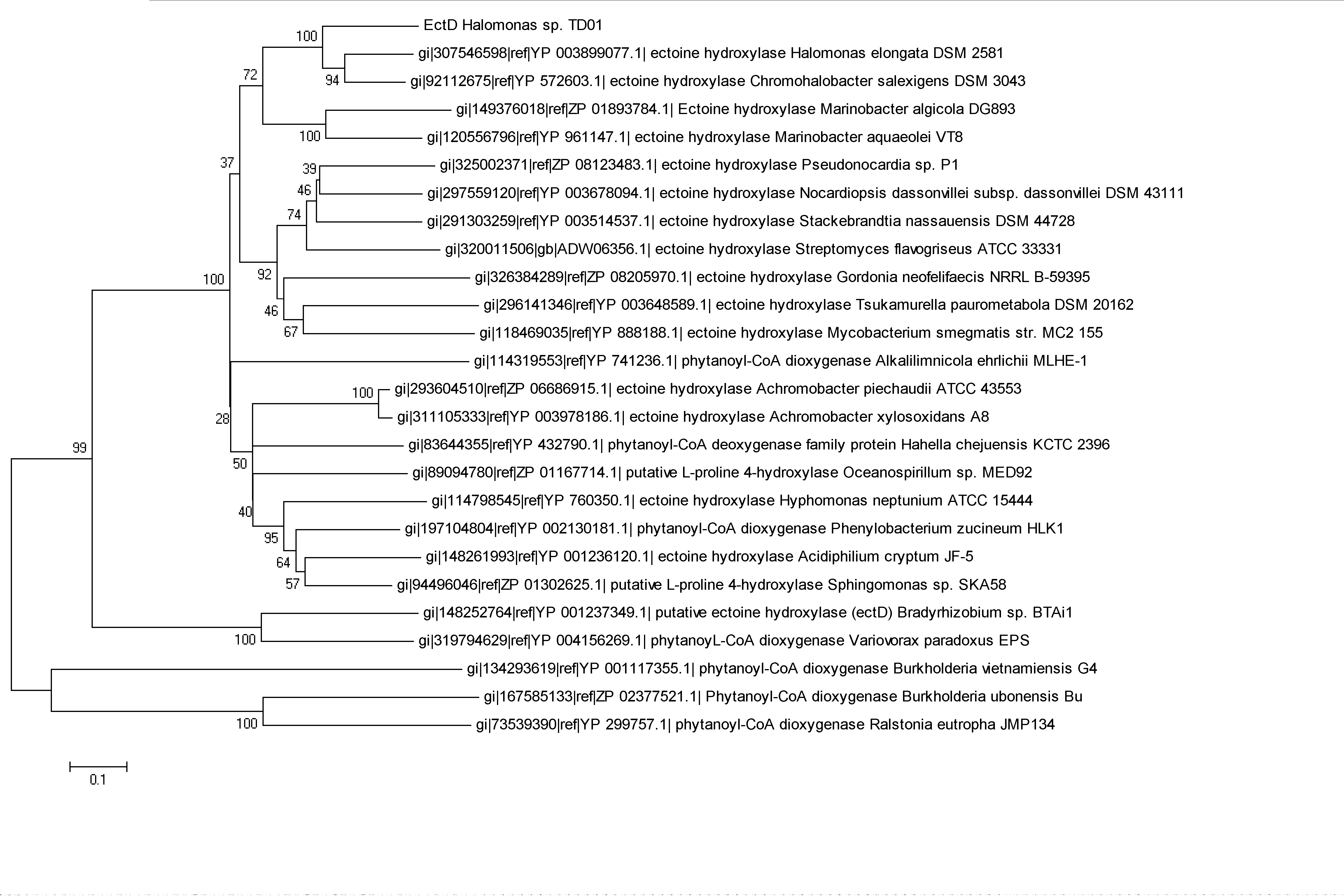


**K**

## Figure S6 - Phylogenetic trees based on the PhaC1 (A), PhaC2 (B), PhaP (C), PhaR (D), PhaZ1 (E), PhaZ2 (F), PhaZ3 (G), EctA (H), EctB (I), EctC (J) and EctD (K) sequences of *Halomonas* sp. TD01 with their homologues of other strains.

The trees were constructed using the neighbor-joining algorithm with MEGA (version 5.03) software. The numbers besides the nodes indicated the bootstrap values based on 500 replications. Bar 0.05/0.1/0.2 substitutions per site were indicated on the graph. The GenBank accession numbers of homologues of PHA and ectoine relevant enzymes were listed in Additional file 1, Table S1.
