# Supplementary material for: Comparative genomics study of polyhydroxyalkanoates (PHA) and ectoine relevant genes from Halomonas sp. TD01 revealed extensive horizontal gene transfer events and co-evolutionary relationships
Source: Microb Cell Fact. 2011 Nov 1;10:88. doi: 10.1186/1475-2859-10-88 (PMC3227634; doi:10.1186/1475-2859-10-88)
Supplement: Additional file 10 — Table S4 and S5. Locus of putative PHA and osmolytes relevant genes in the genome of Halomonas sp. TD01; Transposon and TEprotein identification through the RepeatMasker and RepeatProteinMasker. [file 1475-2859-10-88-S10.DOC]

**Table S4. Locus of putative PHA and osmolytes relevant genes in the genome of *Halomonas* sp. TD01**

| **Gene** | **Scaffold** | **Start position** | **End position** | **Strand^a^** |
| --- | --- | --- | --- | --- |
| *phaC1* | 2 | 549452 | 551302 | + |
| *phaC2* | 4 | 143901 | 146369 | - |
| *phaZ1* | 2 | 436930 | 438075 | + |
| *phaZ2* | 8 | 34957 | 35808 | + |
| *phaZ3* | 3 | 508172 | 509221 | - |
| *phaP* | 2 | 548808 | 549359 | + |
| *phaR* | 1 | 458920 | 459324 | + |
| *ectA* | 7 | 155369 | 155767 | - |
| *ectB* | 7 | 155853 | 157121 | - |
| *ectC* | 7 | 157248 | 157826 | - |
| *ectD* | 9 | 156174 | 157157 | - |

a, positive (+)/negative (-) strand.

**Table S5.** Transposon and TEprotein identification in the genome of *Halomonas* sp. TD01 through the RepeatMasker and RepeatProteinMasker

| **Scaffold** | **Program** | **Start position** | **End position** | **Length (bp)** | **Strand^a^** | **ID** | **Target** | **Class** |
| --- | --- | --- | --- | --- | --- | --- | --- | --- |
| 16 | RepeatMasker | 1265 | 1325 | 270 | - | TE01 | LIN9_SM 5147 5208 | LINE/R2 |
| 21 | RepeatMasker | 13 | 959 | 487 | + | TE02 | RTAg4 1702 2393 | LINE/R1 |
| 1 | RepeatMasker | 32669 | 32725 | 274 | + | TE03 | SINE2-1_SP 10 66 | SINE/tRNA-Lys |
| 1 | RepeatMasker | 33043 | 33106 | 269 | + | TE04 | SINE2-1_SP 3 66 | SINE/tRNA-Lys |
| 1 | RepeatMasker | 212023 | 212087 | 263 | + | TE05 | Rhin-1 12 77 | SINE/SINE |
| 1 | RepeatMasker | 212088 | 212133 | 235 | + | TE06 | TguSINE1 5 112 | SINE/tRNA-CR1 |
| 1 | RepeatMasker | 480859 | 481893 | 520 | - | TE07 | RTAg4 1667 2605 | LINE/R1 |
| 1 | RepeatMasker | 481879 | 483118 | 407 | + | TE08 | Copia-8-I_DR-int 671 1019 | LTR/Gypsy |
| 1 | RepeatMasker | 542330 | 542366 | 237 | + | TE09 | DIRS-2_SP 303 339 | LTR/DIRS |
| 1 | RepeatMasker | 848291 | 848355 | 254 | - | TE10 | ATCopia62_I-int 2744 2810 | LTR/Copia |
| 2 | RepeatMasker | 313493 | 313547 | 234 | + | TE11 | Gypsy-5-LTR_CR 13 68 | LTR/Gypsy |
| 2 | RepeatMasker | 450968 | 451022 | 233 | + | TE12 | hAT1-3_NV 1194 1252 | DNA/hAT-hAT1 |
| 2 | RepeatMasker | 517111 | 517170 | 231 | + | TE13 | CryIIB 2 61 | SINE/SINE |
| 2 | RepeatMasker | 517309 | 517368 | 231 | + | TE14 | CryIIB 2 61 | SINE/SINE |
| 2 | RepeatMasker | 517514 | 517573 | 231 | + | TE15 | CryIIB 2 61 | SINE/SINE |
| 2 | RepeatMasker | 558842 | 558902 | 257 | + | TE16 | SINEC_old 7 67 | SINE/tRNA-Lys |
| 2 | RepeatMasker | 558844 | 558915 | 250 | + | TE17 | LmeSINE1c 6 77 | SINE/Deu |
| 2 | RepeatMasker | 558986 | 559046 | 257 | + | TE18 | SINEC_old 7 67 | SINE/tRNA-Lys |
| 2 | RepeatMasker | 558988 | 559059 | 250 | + | TE19 | LmeSINE1c 6 77 | SINE/Deu |
| 2 | RepeatMasker | 559107 | 559161 | 247 | + | TE20 | SINEC_old 13 67 | SINE/tRNA-Lys |
| 2 | RepeatMasker | 559162 | 559212 | 227 | + | TE21 | SINE_CP1 143 258 | Unknown/Unknown |
| 2 | RepeatMasker | 650966 | 651025 | 250 | - | TE22 | CryIIB 2 61 | SINE/SINE |
| 2 | RepeatMasker | 657744 | 657790 | 227 | - | TE23 | TE2-4_CR 691 737 | Unknown/Unknown |
| 3 | RepeatMasker | 36532 | 36608 | 229 | + | TE24 | DIRS1a_DR 5875 5953 | LTR/DIRS |
| 3 | RepeatMasker | 93795 | 93882 | 233 | + | TE25 | Penelope2_XT 3095 3180 | LINE/Penelope |
| 3 | RepeatMasker | 662237 | 662292 | 230 | - | TE26 | MuDR-11N_VV 3304 3365 | DNA/MuDR |
| 4 | RepeatMasker | 20972 | 21075 | 228 | + | TE27 | ERV2X1A-I_ML-int 6439 6539 | LTR/ERVK |
| 4 | RepeatMasker | 49483 | 49609 | 245 | - | TE28 | Gypsy-71-I_NV-int 595 725 | LTR/Gypsy |
| 4 | RepeatMasker | 55679 | 55694 | 228 | - | TE29 | SINE2-2_SP 13 85 | SINE/tRNA-Lys |
| 4 | RepeatMasker | 55695 | 55750 | 233 | - | TE30 | Rhin-1 12 67 | SINE/SINE |
| 4 | RepeatMasker | 55778 | 55873 | 233 | - | TE31 | Rhin-1 8 96 | SINE/SINE |
| 4 | RepeatMasker | 129162 | 129229 | 230 | + | TE32 | L1BM 1831 1897 | LINE/Jockey |
| 4 | RepeatMasker | 201518 | 201592 | 239 | - | TE33 | BEL9-I_DR-int 2083 2153 | LTR/Pao |
| 4 | RepeatMasker | 207390 | 207448 | 239 | - | TE34 | Gypsy8-SM_I-int 8921 8980 | LTR/Gypsy |
| 4 | RepeatMasker | 225483 | 225517 | 234 | + | TE35 | DIRS-9_NV 3157 3192 | LTR/DIRS |
| 5 | RepeatMasker | 100679 | 100739 | 228 | - | TE36 | CEREBA_HV-int 1526 1583 | LTR/Gypsy |
| 5 | RepeatMasker | 100693 | 100763 | 243 | + | TE37 | Gypsy-80-I_ZM-int 5924 5979 | LTR/Gypsy |
| 5 | RepeatMasker | 104023 | 104118 | 233 | + | TE38 | ID_Rn1 4 95 | SINE/ID |
| 7 | RepeatMasker | 82572 | 82630 | 233 | - | TE39 | Merlin1_HS 762 828 | DNA/Merlin |
| 7 | RepeatMasker | 121128 | 121162 | 232 | - | TE40 | hAT-1_HV 471 507 | DNA/hAT-Ac |
| 7 | RepeatMasker | 199408 | 199473 | 238 | - | TE41 | ID4 9 74 | SINE/ID |
| 7 | RepeatMasker | 199529 | 199594 | 238 | - | TE42 | ID4 9 74 | SINE/ID |
| 8 | RepeatMasker | 7864 | 7955 | 233 | - | TE43 | Gypsy2-HV_I-int 4960 5057 | LTR/Gypsy |
| 10 | RepeatMasker | 41733 | 41780 | 241 | + | TE44 | Tc1-17a_Xen 257 304 | DNA/TcMar-Tc1 |
| 10 | RepeatMasker | 52555 | 52607 | 230 | - | TE45 | Kolobok-1_XT 178 232 | DNA/Kolobok |
| 9 | RepeatMasker | 72120 | 72179 | 227 | - | TE46 | SINEC_old 11 70 | SINE/tRNA-Lys |
| 9 | RepeatMasker | 72247 | 72306 | 227 | - | TE47 | SINEC_old 11 70 | SINE/tRNA-Lys |
| 9 | RepeatMasker | 104521 | 104591 | 276 | + | TE48 | Copia4_LTR_MT 270 340 | LTR/Copia |
| 23 | RepeatProteinMask | 10 | 627 | 210 | - | TP01 | IS30_tp 59 263 | DNA/IS |
| 1 | RepeatProteinMask | 116073 | 116924 | 494 | + | TP02 | IS3_tp 1 285 | DNA/IS |
| 1 | RepeatProteinMask | 217504 | 218451 | 473 | + | TP03 | IS30_tp 59 374 | DNA/IS |
| 1 | RepeatProteinMask | 233953 | 234588 | 88 | + | TP04 | IS30_tp 49 269 | DNA/IS |
| 1 | RepeatProteinMask | 234577 | 234801 | 155 | + | TP05 | IS30_tp 297 371 | DNA/IS |
| 1 | RepeatProteinMask | 238348 | 238704 | 100 | + | TP06 | IS3_tp 123 238 | DNA/IS |
| 1 | RepeatProteinMask | 604799 | 605182 | 35 | + | TP07 | 45368562_AD 12 140 | DNA/TcMar-IS885 |
| 2 | RepeatProteinMask | 181223 | 182074 | 494 | - | TP08 | IS3_tp 1 285 | DNA/IS |
| 2 | RepeatProteinMask | 323237 | 323926 | 140 | + | TP09 | IS150_insB 55 295 | DNA/IS |
| 4 | RepeatProteinMask | 109389 | 110078 | 140 | - | TP10 | IS150_insB 55 295 | DNA/IS |
| 6 | RepeatProteinMask | 119400 | 119882 | 107 | - | TP11 | MurERV4_pro 26 169 | LTR/ERVK |
| 6 | RepeatProteinMask | 119499 | 119864 | 115 | - | TP12 | FIV-C_pol 726 841 | LTR/Lenti |
| 6 | RepeatProteinMask | 263673 | 263951 | 270 | + | TP13 | IS30_tp 288 380 | DNA/IS |
| 7 | RepeatProteinMask | 86994 | 87845 | 494 | + | TP14 | IS3_tp 1 285 | DNA/IS |
| 8 | RepeatProteinMask | 25178 | 25456 | 270 | + | TP15 | IS30_tp 288 380 | DNA/IS |

a, positive (+)/negative (-) strand.
